# Neural Crest-Derived Stem Cell Secretomes and Extracellular Vesicles Disrupt Glioblastoma through Dual-Pathway Inflammatory Rebalancing

**DOI:** 10.1007/s12015-026-11133-5

**Published:** 2026-04-28

**Authors:** Atiyeh Asadpour, Nagihan Ozsoy, Leah Napier, Kathryn Cox, Sarah Needs, Kirk A. Taylor, Helen Brown, Augustas Pivoriūnas, Phil Stephens, Phil Dash, Graeme S. Cottrell, Darius Widera

**Affiliations:** 1 Stem Cell Biology and Regenerative Medicine Group, School of Pharmacy, University of Reading, Reading, UK; 2 School of Biological Sciences, University of Reading, Reading, UK; 3 School of Pharmacy, University of Reading, Reading, UK; 4 School of Dentistry, College of Biomedical & Life Sciences, Cardiff University, Cardiff, UK; 5 Department of Stem Cell Biology, State Research Institute Centre for Innovative Medicine, Vilnius, Lithuania; 6 Cellular and Molecular Neuroscience, School of Pharmacy, University of Reading, Reading, UK

**Keywords:** Glioblastoma, Neural crest-derived stem cells, Stem cell secretome, Extracellular vesicles, NF-kappaB signalling, IRF3 signalling, Temozolomide chemosensitisation, Organotypic slice cultures

## Abstract

Glioblastoma multiforme is characterised by resistance to conventional treatments via sustained pro-inflammatory signalling. This study investigated whether secretomes and small extracellular vesicles (sEVs) from human oral mucosa neural crest-derived stem cells (NCSCs) could disrupt multiple glioblastoma features by rebalancing pro- and anti-inflammatory pathways. NCSCs were characterised, and their secretomes/sEVs isolated, and analysed using a range of state-of-the-art methods. Anti-tumour effects were evaluated using multiple glioblastoma cell lines (U251, U373, U87) through viability, proliferation, migration, and tumourigenicity assays. Mechanistic studies employed dual nuclear factor-κB (NF-κB) and quadruple NF-κB/interferon regulatory factor 3 (IRF3) reporter systems, cytokine arrays, and immunocytochemistry. Therapeutic potential was assessed using temozolomide (TMZ) chemosensitivity assays and organotypic mouse brain slice models. NCSC secretomes and sEVs demonstrated consistent anti-glioblastoma activity across all functional assays. Both products potently suppressed NF-κB activation across multiple pro-inflammatory stimuli whilst simultaneously enhancing IRF3 nuclear translocation and transcriptional activity. This dual pathway modulation reprogrammed glioblastoma cytokine secretion towards anti-inflammatory profiles, and inhibited tumourigenicity in both 3D culture and ex vivo brain slice models. Notably, secretomes and sEVs enhanced the efficacy of TMZ, reducing colony size compared to monotherapy, without compromising tissue viability. These findings demonstrate the first evidence of dual NF-κB/IRF3 pathway rebalancing by NCSC-derived products in glioblastoma. The simultaneous suppression of tumour-promoting inflammation whilst enhancing anti-tumour immune signalling represents a novel therapeutic paradigm that addresses multiple resistance mechanisms simultaneously. This strategy redefines stem cell-based therapy for glioblastoma by integrating immunomodulation with chemosensitisation in a single, Good Manufacturing Practice (GMP)-compatible product.

**Graphical Abstract:**

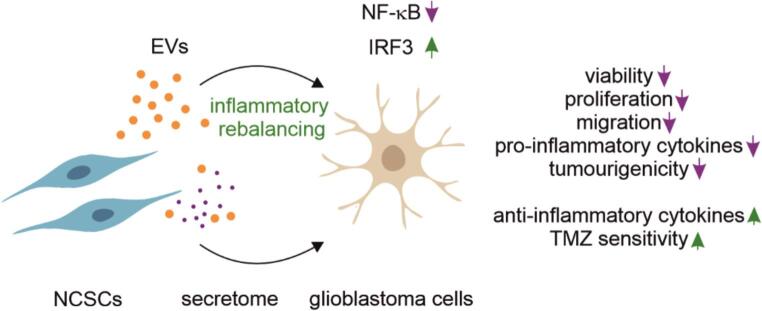

**Supplementary Information:**

The online version contains supplementary material available at 10.1007/s12015-026-11133-5.

## Introduction

Glioblastomas are the most aggressive and lethal primary brain tumours in adults, characterised by a median survival of merely 14–16 months despite maximal therapeutic intervention [1, 2]. This poor prognosis results from a highly heterogeneous and immunosuppressive microenvironment in glioblastomas, where neoplastic cells systematically recruit resident microglia, infiltrating macrophages, and astrocytes to sustain proliferation, diffuse invasion, and therapeutic resistance [1, 3]. The complex interplay between glioblastoma cells and their surrounding milieu creates substantial barriers to effective treatment, highlighting the need for novel treatment options that can simultaneously target multiple pathways within the signalling networks regulating the tumour behaviour.

Inflammation is one of the emerging hallmarks of cancer [4]. Briefly, chronic inflammatory conditions create a microenvironment that supports malignant transformation, tumour progression, and metastasis via sustained activation of pro-oncogenic signalling pathways. The transcription factor NF-κB serves as the central mediator and master regulator connecting tumour-promoting inflammation to cancer via regulation of pro-inflammatory cytokines, survival factors, and tumour-promoting genes [5]. Constitutive activity of NF-κB is pivotal in the pro-tumorigenic microenvironment in glioblastomas, where it drives stem-like characteristics, migration, angiogenesis, and resistance to both radiotherapy and chemotherapy [6–9]. However, its pharmacological inhibition in glioblastoma is not a viable therapeutic strategy due to severe systemic toxicities, as demonstrated by the early termination of a sulfasalazine clinical trial where four patients developed grade 4 toxicity and two died. This suggests that balanced modulation rather than global inhibition of inflammatory signalling may offer safer and more effective therapeutic approaches [6].

Contrary to NF-κB, IRF3 acts as a negative regulator of glioma invasion and proliferation. Enforced IRF3 expression or its pharmacological activation down-regulates the expression of pro-invasive extracellular-matrix genes, reduces migration, and limits proliferation of patient-derived glioblastoma cells and xenografts, whereas a loss of IRF3 has the opposite effect [10, 11].

Thus, a simultaneous manipulation of these opposing signalling pathways represents an attractive therapeutic option for modulating glioblastoma behaviour.

Adult NCSCs are a population of multipotent stem cells that persist within the mammalian craniofacial compartment, retaining some of the developmental plasticity of their embryonic counterparts. NCSCs can differentiate into ectodermal derivatives, such as neurons, and mesodermal progeny, including osteoblasts, making them promising candidates for regenerative medicine [12–16]. We demonstrated that transplantation of human nasal NCSCs in a rat model of Parkinson`s disease leads to behavioural recovery and restored dopaminergic neuron population [17]. Despite this beneficial pre-clinical outcome, no human dopaminergic neurons were found in the substantia nigra, suggesting a paracrine mode of action. Indeed, the paracrine output of NCSCs has emerged as a key mediator of their regenerative effects [16, 18–21].

Stem cell secretomes contain cytokines, chemokines, growth factors, nucleic acids, and peptides, alongside EVs [22]. The composition of secretomes varies significantly depending on the cellular origin and the age of the individual donor [23].

EVs are small particles enclosed within a lipid bilayer that all cells release into their surroundings. EVs vary in their dimensions, contents, and biological roles, leading to their classification into distinct categories. The classification system distinguishes between sEVs, typically smaller than 200 nanometres in diameter, and large EVs, which exceed 200 nanometres [24]. EVs facilitate the transfer of proteins, nucleic acids, and lipids between different cells. This subsequently modulates various processes in both the cells that secrete the vesicles and those that receive them. The delivery of EV-cargo can occur through several mechanisms: endocytosis, direct membrane fusion, or micropinocytosis [25, 26]. Additionally, EVs may act through interactions between membrane-associated ligands present on their surface and corresponding receptors on target cells [27, 28].

The paracrine mode of action of NCSCs offers several advantages over whole-cell therapeutic approaches. Secretomes and EVs derived from NCSCs are non-tumorigenic, display low immunogenicity, and can be standardised under GMP conditions [10, 29–31] making NCSC-derived biologics promising candidates for cell-free therapies.

NCSCs and mesenchymal stromal cells produce secretomes and sEVs that potently suppress NF-κB signalling, as demonstrated by their ability to prevent TNF-α, IL-1β, and IL-6-induced NF-κB activation in reporter cell lines [32–34].

Interestingly, it has been demonstrated that *Plasmodium falciparum*-derived EVs can activate IRF3 to induce the transcription of host genes [35]. Moreover, bacterial sEVs have been shown to activate the upstream kinase TBK1, which phosphorylates and activates IRF3 [36]. Mechanistically, both studies suggested that activation of IRF3 was caused by the delivery of pathogen-associated molecular patterns as cargo of sEVs.

In 2022, Popli and colleagues showed that virally induced activation of IRF3 can directly inhibit nuclear translocation of NF-κB p65 in human and mouse cells [37]. A more recent study demonstrated that Tumour Necrosis Factor Receptor-Associated Factor 6 promotes nuclear translocation of p65 via suppressing IRF3 in gastric cancer cells [38]. Together, these findings suggest that IRF3 and NF-κB are regulating or counterbalancing each other.

We hypothesised that NCSC-derived secretomes and sEVs can reduce the viability and the levels of sustained proliferation, diffuse invasion, tumorigenicity, and therapy resistance of glioblastoma cells by shifting the balance between NF-κB and IRF3. We investigated whether secretomes and sEVs derived from adult human NCSCs could disrupt multiple glioblastoma features through dual-pathway inflammatory rebalancing. We examined the effects of NCSC-derived products on glioblastoma cell viability, proliferation, migration, and tumourigenicity using glioma cell lines and advanced reporter systems. We evaluated the mechanistic basis of any observed effects by assessing NF-κB and IRF3 pathway modulation, and cytokine profile changes. Finally, we tested the therapeutic potential of these products in combination with standard chemotherapy using both 3D culture systems and clinically relevant organotypic brain slice models.

## Methods

### Cell Culture

Human glioblastoma cell lines (U251-MG, U87-MG, and U373-MG; all ECACC) were cultured in high-glucose DMEM supplemented with 10% EV-depleted foetal bovine serum (FBS, Lot: 1693724), and L-glutamine (200 mM) (all from Sigma-Aldrich). NF-κB dual reporter cells (U251-NF-κB-GFP-Luc) [39] were cultivated in supplemented with 10% FBS, L-glutamine (200 mM), and puromycin (1 µg/ml) while NF-κB/IRF3 quadruple reporter cells (U251-NF-κB-IRF3-Luc) were cultured in DMEM supplemented with 10% FBS, L-glutamine, puromycin (1 µg/ml), and blasticidin (5 µg/ml).

Immortalised oral mucosa-derived NCSCs [40, 41], were cultured in DMEM/F12 medium (Sigma-Aldrich), supplemented with 10% FBS, L-glutamine, and 10 ng/mL recombinant human FGF-2 (PeproTech).

### Secretome Generation

Cells were cultured as adherent monolayers and grown to approximately 70% confluence in T175 cell culture flasks and secretomes were isolated as described earlier [42].

### Isolation of EVs

EVs were isolated from secretomes using ExoSpin columns (Cell Guidance Systems) according to the manufacturer’s instructions. EV pellets were resuspended in sterile PBS containing 25mM D-(+)-trehalose dihydrate and 0.2% human serum albumin (both Sigma Aldrich) and stored at − 80 °C until further use.

### Nanoparticle Tracking Analysis (NTA)

The concentration and size of EVs were evaluated using nanoparticle tracking analysis as described in [43], using an NS500 instrument (Malvern).

### Transmission Electron Microscopy (TEM)

A drop of re-suspended EV pellet in PBS was placed on parafilm and absorbed onto mesh carbon-coated copper grids for 5 min. Samples were fixed with 1% glutaraldehyde for 1 h, washed four times (each for 30 s), and subjected to negative staining for 2 min with 1% uranyl acetate. After air drying, grids were examined using a Zeiss 906 transmission electron microscope (Zeiss). EV size was quantified by manually measuring the diameters of EV populations using Fiji software [44].

### SDS-PAGE and Western Blotting

EV samples and whole-cell lysates were resolved by SDS-PAGE on 15% acrylamide gels, followed by transfer of proteins to PVDF membranes (Immobilon-P, Millipore). Membranes were incubated (1 h, room temperature (RT)) in blocking buffer consisting of 1×PBS, 0.1% Tween^20^, and 5% non-fat milk powder. For detection, membranes were incubated (overnight, 4 °C) with primary antibodies against CD9 (1:1000, clone EPR23105, Abcam), CD63 (1:1000 dilution, clone EPR5702, Abcam), and CD81 (1:1000 dilution, clone EPR4244, Abcam) in blocking buffer. Membranes were washed (30 min, PBS with 0.1% Tween^20^) and incubated with donkey anti-rabbit IgG-HRP (1:10,000, 1 h, RT) in blocking buffer. Immunoreactive bands were visualised using enhanced chemiluminescence (Bio-Rad), and densitometry was performed with an ImageQuant-RT ECL imaging system (GE Healthcare) using ImageQuant TL software for analysis.

### EV-labelling and Uptake Assays

U251 cells (50,000 cells/well) were cultured on coverslips in 12-well plates until reaching approximately 50% confluence, washed three times with DMEM, and incubated (30 min, 37 °C) with CellTracker™ Red CMTPX (1:2000; Invitrogen).

EVs (1 × 10⁶ cells) were labelled by incubation with PKH67 (Sigma-Aldrich, 6 µL, 5 min, RT). CellTracker™-stained U251 cells were incubated with PKH67-labelled EVs (3 h, 37 °C). Cells were then fixed in 4% paraformaldehyde (PFA, 15 min, RT), washed three times with PBS, and counterstained with DAPI. Imaging was performed using an Operetta CLS high-content imaging system (Revvity) in confocal mode.

### Viability Assays

U251 cells were exposed to 10 ng/ml TNF-α (Peprotech), 2% (v/v) secretome, or 2% (v/v) EVs or a combination of TNF-α and secretomes or EVs for 72 h. Cell viability was assessed using the Cell Proliferation Kit II (Sigma-Aldrich) according to the manufacturer’s instructions. Absorbance of the XTT metabolite was measured at 490 nm (excitation) and 650 nm (reference) using a SpectraMax i3x (Molecular Devices) after 4 h.

### Proliferation Assays

U251 cells (5,000 cells/well) were seeded in 24-well plates and exposed to 10 ng/mL TNF-α, 2% (v/v) secretome, 2% (v/v) EVs, or combinations of TNF-α with either secretome or EVs, followed by incubation at 37 °C for 72 h. At designated time points, cell enumeration was performed using a CytoSMART automatic cell counter (Corning). To verify the results of total cell number determination, ICC for Ki67 was performed. U251 cells (5,000 cells/well) were seeded in 96-well plates, starved, and treated with 2% (v/v) secretome, 2% (v/v) EVs, or combinations of TNF-α and secretome or EVs for 24 h, followed by an additional 30 min treatment with TNF-α (10 ng/mL, 37 °C). Following fixation with 4% PFA (15 min, RT), cells were blocked (30 min, RT) in PBS containing 5% horse serum (Sigma-Aldrich) and 0.02% Triton X-100 (Apollo Scientific Ltd.). Cells were then subjected to immunocytochemistry for Ki67 as described below.

### Migration Assay

U251 cells (20,000 cells/well) were seeded in 12-well plates and incubated in standard growth medium. After reaching approximately 70% confluency, a vertical scratch was created across the cell monolayer in each well using a P200 pipette tip. The culture medium was removed, and cells were gently washed once with 1 mL pre-warmed DMEM. Fresh complete medium supplemented with either 2% (v/v) vehicle control, 2% (v/v) whole secretome, EVs, 10 ng/mL TNF-α, or combinations of TNF-α with secretome or EVs was added to each well.

Cell migration was monitored using a Nikon TiE imaging system (Nikon), with images captured every 20 min over 21 h. Gap closure analysis between 0 h and 21 h was quantified as percentage of wound closure using Fiji software.

### Immunocytochemistry (ICC)

Cells were fixed in ice-cold PBS containing 4% PFA (15 min, RT). Permeabilisation and blocking were performed using 0.02% Triton X-100 and 5% horse serum (30 min, RT). For immunocytochemical analysis, fixed and permeabilised cells were incubated (1 h, RT) with the appropriate primary antibodies: monoclonal mouse anti-NF-κB p65 (clone F-6, 1:100; SC-8008 #D-1919), monoclonal mouse anti-CD9, anti-CD63, and anti-CD81 (all 1:100; all Santa Cruz Biotechnology), monoclonal rabbit anti-IRF3 (1:100, clone SD2062, Life Technologies), polyclonal rabbit anti-pIRF3 (1:100, clone 720012, Invitrogen), and monoclonal mouse anti-Ki67 (1:300, clone AFFN-Ki67-3E6, DSHB).

Samples were then washed with PBS (5 min, RT), and incubated with Alexa Fluor-conjugated secondary antibodies (1 h, RT). Secondary antibodies included donkey anti-rabbit Alexa488, Alexa555, and Alexa647, donkey anti-mouse Alexa488, Alexa555, and Alexa647 (all 1:300; Invitrogen).

Cell nuclei were stained using 4’,6-diamidino-2-phenylindole (DAPI) solution (1 mg/mL, 1:2000, Sigma-Aldrich, UK), and cells were washed twice with PBS (5 min) and once with sterile distilled water (1 min). Samples were visualised using an Operetta CLS high-content imaging system.

### Translocation of NF-κB and IRF3

To assess the nuclear translocation of NF-κB p65, U87-MG, U251-MG, and U373-MG cells were pre-cultured for 24 h, then serum-starved (3 h). The medium was then replaced with medium containing FBS and either vehicle control, 2% (v/v) secretome, or 2% (v/v) EVs for 24 h. Cells were then treated with TNF-α (10 ng/mL, 30 min), or combinations of TNF-α with secretome or EVs. Following treatment, cells were washed with ice-cold PBS (5 min), fixed and NF-κB subunit p65 localised as described above.

To monitor the subcellular localisation of IRF3, U251 cells were pre-cultured for 24 h and serum-starved for 3 h. The medium was then replaced with medium containing FBS, vehicle control, 2% (v/v) secretome, or 2% (v/v) EVs, and cells were incubated for 24 h. Following incubation, cells were fixed and total IRF3 and phosphorylated IRF3 localised as described above.

Imaging was performed using the Operetta CLS high-content imaging system (Revvity). Unsupervised analysis of the subcellular localisation of p65, IRF3, and phosphorylated IRF3 was conducted using the Harmony software suite (Revvity). Relative fluorescence=F_exp_-F_min_)/(F_max_-F_min_)×100. F_exp_ (experimental fluorescence value), F_max_ (maximum fluorescence), F_min_ (background fluorescence).

### Luciferase Assays

NF-κB activity was evaluated using the Bright-Glo^®^ Luciferase Assay System (Promega), performed in accordance with the manufacturer’s instructions. U251 dual NF-κB reporter cells (1 × 10⁴ cells/well) were seeded in 96-well plates and cultured for 24 h, then serum starved (medium containing 0.1% bovine serum albumin (BSA), 4 h, 37 °C). Cells were returned to complete medium containing FBS, pre-treated with secretome (2% v/v) for 4 h, and stimulated with TNF-α (10 ng/mL), interleukin-1β (IL-1β, 10 ng/ml), or interleukin-6 (IL-6, 50 ng/ml), either alone or in combination with secretome or EVs. Cultures were maintained for an additional 48 h. For luminescence detection, culture medium was replaced with DMEM without phenol red (Sigma-Aldrich). Bright-Glo^®^ substrate was added, and luminescence signals were measured using a SpectraMax i3x microplate reader.

For simultaneous assessment of NF-κB and IRF3 activity, U251 quadruple NF-κB/IRF3 reporter cells (1 × 10⁴ cells/well) harbouring both response elements were processed using the Dual-Glo^®^ Luciferase Assay System (Promega), following the manufacturer’s protocol. After serum starvation (4 h), cells were treated with either secretome or EVs (2% v/v) for 2 h, followed by stimulation with TNF-α (10 ng/mL) ± secretome or EVs for 48 h. Prior to the assay, media were replaced with DMEM without phenol red (Sigma-Aldrich). Firefly luciferase activity was recorded after addition of Dual-Glo^®^ Luciferase Reagent and subsequently quenched with Dual-Glo^®^ Stop & Glo^®^ Reagent to facilitate measurement of *Renilla* luciferase. All luminescence readings were acquired using the SpectraMax i3x microplate reader. Relative luminescence=(L_exp_-L_min_)/(L_max_-L_min_)×100. L_exp_ (experimental luminescence value), L_max_ (maximum luminescence), L_min_ (background luminescence).

### Human Cytokine Array

Cytokine profiling was performed using the Human Cytokine Antibody Array Membrane (42 targets; Abcam) according to the manufacturer’s instructions. Briefly, U251 cells (1 × 10^6^) were exposed to TNF-α (10 ng/mL), secretome (2% (v/v)), EVs (2% (v/v)), or combinations of TNF-α with secretome or EVs for 24 h, then lysed. Membranes were pre-blocked with 1 x blocking buffer, and 1 mL of treated lysate was added to each membrane and incubated (overnight, 4 °C) with gentle agitation. After washing, membranes were incubated (overnight, 4 °C) with biotin-conjugated detection antibodies.

Following additional washes, membranes were incubated (overnight, 4 °C) with HRP-conjugated detection antibody. Chemiluminescent detection reagents were applied, and membrane signals were captured by imaging with an ImageQuant LAS 4000 system (GE Healthcare). Spot intensities were quantified using Fiji software and normalised to positive control spots for semi-quantitative analysis.

### Tumourisphere Assays

Single U251 cell suspensions were seeded (2,000 cells in ultra-low attachment 24-well plates (Corning) in 1 mL) in medium composed of DMEM/F12 (Sigma-Aldrich) supplemented with 1× B27 supplement (Life Technologies, UK), 20 ng/mL epidermal growth factor (EGF; Qkine), and 20 ng/mL basic fibroblast growth factor (FGF-2; Qkine) Experimental groups were exposed to TNF-α (10 ng/mL), 2% (v/v) secretome, 2% (v/v) EVs, or combinations of TNF-α with secretome or EVs. Tumourispheres were cultured for 14 days. For medium changes, spheres were collected by centrifugation at 300 *g*.

Images were captured using an EVOS FL digital inverted microscope (Life Technologies), and sphere number and size were determined using Fiji software.

### Soft Agar Assays

12-well plates were prepared with 0.5% base agar (Noble Agar; Sigma Aldrich), then overlayed with 0.3% top agar containing 5 × 10³ U251 cells and supplemented with TNF-α (10 ng/mL), 2% (v/v) secretome, 2% (v/v) EVs, or combinations of TNF-α with secretome or EVs.

Cultures were maintained for 21 days. Colonies were stained with 0.01% crystal violet for 30 min at room temperature. Images were captured using an EVOS FL digital microscope (Life Technologies), and colony number and size were quantified for each condition in the entire well using Fiji software.

### Chemosensitivity Assays

U251 cells (1,250 cells/well) were cultivated in 96-well plates. Plates were incubated for 24 h, then cells were pre-treated with 2% (v/v) secretome, 2% (v/v) EVs. After 2 days, cells were exposed to TMZ (20 µM, Sigma Aldrich, UK) for 5 days in addition to untreated controls. Cell viability was determined using the XTT assay as described above.

In addition, soft agar assays were conducted in the presence of TMZ, 2% (v/v) secretome, 2% (v/v) EVs, and combinations of TMZ with secretome or EVs, as described above.

### Tissue Procedures and Organotypic Culture Setup

All tissue procedures were conducted in accordance with UK ethical guidelines for the care and use of laboratory animals as mandated by the Animals (Scientific Procedures) Act 1986. All steps involving cells were performed under aseptic conditions in a Class II safety cabinet.

Six-well plates (Corning, Ewloe, UK) were prepared with standing cell culture membrane inserts (PICM03050, Millipore, UK). Each well contained 1 mL Neurobasal-A medium (10888022, Life Technologies) supplemented with 25% Hank’s Balanced Salt Solution (HBSS; 14175095, Gibco), 10% FBS (A5256701, Life Technologies), 2% B-27 Supplement (17504044, Life Technologies), 1% penicillin–streptomycin (10,000 U/mL; 15140122, Life Technologies), and 1% GlutaMAX (35050061, Life Technologies). All surfaces and instruments were cleaned with 70% ethanol; dissection tools were further sterilised under ultraviolet light in 70% ethanol for 30 min prior to use.

Brains were obtained from pre-weaned C57BL/6J mice (postnatal day 18–20), anaesthetised with isoflurane until loss of toe-pinch reflex, sacrificed by cervical dislocation, and decapitated. Brains were rapidly removed and placed in ice-cold sucrose-based artificial cerebrospinal fluid (saCSF) containing 189 mM sucrose, 10 mM glucose, 26 mM NaHCO₃, 3 mM KCl, 5 mM MgSO₄·7 H₂O, 0.1 mM CaCl₂, and 1.25 mM NaH₂PO₄, continuously oxygenated with 95% O₂/5% CO₂.

Cerebella and brain stems were excised, hemispheres separated and affixed midline-down with cyanoacrylate glue onto the stage of a vibratome (Leica VT1200S) submerged in ice-cold, carbogenated saCSF. Brains were sectioned sagittally at 0.1 mm/s until the hippocampus became visible, then sliced into sections of 350 μm thickness. Slices were transferred to a holding chamber containing carbogen-bubbled artificial cerebrospinal fluid (aCSF: 124 mM NaCl, 3 mM KCl, 24 mM NaHCO₃, 1.25 mM NaH₂PO₄, 1 mM MgSO₄·7 H₂O, 2 mM CaCl₂, 10 mM glucose) and incubated at 37 °C for one hour to recover.

Two viable hippocampal slices were placed onto each membrane insert and maintained at 37 °C. Medium was changed every two days. Slices demonstrating macroscopic damage or non-viability were excluded prior to and during culture. All slices were cultured for 5 days prior to subsequent treatments or cell addition.

### Lactate Dehydrogenase (LDH) Cytotoxicity Assay

Brain slices prepared as described above were exposed to TNF-α (10 ng/ml), 2% (v/v) secretomes, or EVs. Cellular cytotoxicity was assessed on days in vitro (DIV) 6, 8, 12, and 14 using the CyQUANT™ LDH Cytotoxicity Assay (Invitrogen), ensuring consistent sampling intervals post-treatment. For each time point, a 96-well plate was prepared in triplicate with the following conditions: fresh culture medium (negative control), medium from cultures treated with 0.5% Triton X-100 for 1 h (positive control) and spent media from each treatment condition. For each sample, 50 µL was transferred to a well, followed by 50 µL reaction mixture. After incubation (30 min, RT), protected from light, the reaction was stopped, and absorbance was measured at 490 nm.

### Cell Transplantation

On day 6 in vitro (DIV), co-cultures were initiated by adding 20,000 U251 glioma cells (in 50 µL supplemented Neurobasal-A medium) directly onto each slice, ensuring gradual and complete adsorption. Slices were then allocated to either control conditions or treated with 2% (v/v) NCSC secretome or EVs. Treatments and culture medium were replenished every two days. On DIV 8, half the slices from each group received additional treatment with TNF-α (10 ng/mL); each condition was performed in triplicate.

### Immunohistochemistry

Slices were fixed with 4% PFA (30 min, RT). To permeabilise and block nonspecific binding, sections were pre-incubated for 1 h in 1× Tris-buffered saline (TBS) containing 0.5% Triton X-100 and 5% horse serum. Subsequently, slices were incubated overnight at 4 °C with monoclonal mouse anti-human nestin (R&D Systems) in a 24-well plate on a platform rocker (Stuart, Str 6) at 5 rev/min.

After three washes with 1× TBS, slices were incubated overnight at 4 °C with donkey anti-mouse Alexa 488 (Invitrogen). Nuclear counterstaining was performed with DAPI (2 h). After two washes, once with TBS and once with double-distilled water, sections were placed on microscope slides for imaging. Confocal imaging was carried out using a Nikon A1R microscope (Nikon), and images were analysed using Fiji.

### Statistical Analysis

Statistical analyses were performed using GraphPad Prism software (version 8.4.3; GraphPad, San Diego, CA, USA). Data were compared using either Student’s t-test (two-tailed, 95% confidence interval) or one-way analysis of variance (ANOVA) with Tukey’s post hoc test, as appropriate. At least three independent experiments were performed, each with technical triplicates. A p-value of < 0.05 was considered statistically significant.

## Results

### Characterisation of NCSCs and sEV Marker Expression

NCSCs were characterised using ICC. Confocal microscopy revealed robust expression of the NCSC marker nestin with a characteristic filamentous cytoplasmic staining pattern, and elongated morphology consistent with NCSCs (Figure S1).

**Fig. 1:**
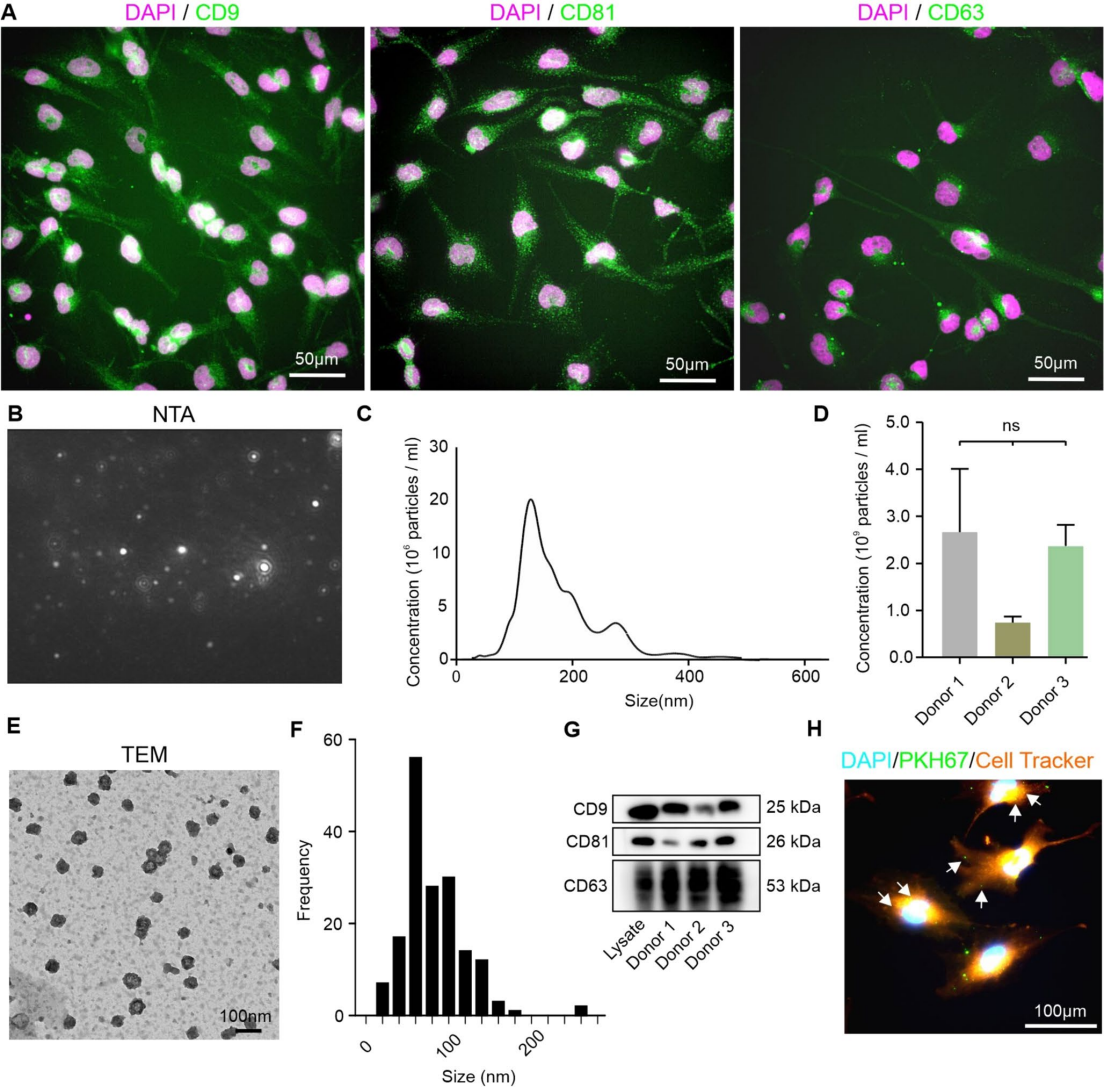
Characterisation of NCSC-derived small extracellular vesicles (sEVs). **A** Immunocytochemical staining showing expression of classical sEV markers CD9, CD81, and CD63 (green) in NCSCs with distinct punctate staining patterns. CD9 and CD81 show more abundant expression compared to CD63. Bars: 50 μm. **B** Representative nanoparticle tracking analysis (NTA) traces. **C** Size distribution histogram revealed a characteristic peak with particles averaging ~ 60 nm diameter. **D** Quantification of sEV concentration (~ 2 × 10¹⁰ particles/mL) across three donor samples showed consistent production with no significant differences between donors. **E** Transmission electron microscopy (TEM) images showed typical cup-shaped morphology of isolated sEVs following negative staining. Scale bar, 100 nm. **F** Size distribution analysis from TEM images confirming predominant vesicle population in 40–80 nm range. **G** Western blot analysis confirming expression of canonical sEV markers CD63 (~ 53 kDa), CD81 (~ 26 kDa), and CD9 (~ 25 kDa) across all three donor samples. **H** Spinning disc confocal microscopy images demonstrated uptake of PKH67-labelled sEVs (green) by CellTracker Red-labelled U251 glioblastoma cells (orange), demonstrating cellular internalisation and membrane binding. Nuclei are counterstained with DAPI (cyan). Examples of EVs are highlighted by arrows

NCSCs were confirmed to express the classical sEV markers CD9, CD81, and CD63 (Fig. 1A), with distinct punctate staining patterns for all three tetraspanins.

### Characterisation of NCSC sEVs

NTA demonstrated that NCSCs actively secreted sEVs at a concentration of 2 × 10¹⁰ particles/ml (Fig. 1B). Analysis revealed a characteristic peak with particles averaging approximately 60 nm in diameter, consistent with the expected size range for sEVs (Fig. 1C). Concentration analysis showed no significant differences between donors (Fig. 1D).

TEM analysis further confirmed the typical cup-shaped morphology characteristic of sEVs (Fig. 1E). The size distribution analysis verified the NTA findings with a predominant population of vesicles in the 40–80 nm range (Fig. 1F).

Western blot analysis confirmed the presence of sEV markers CD63, CD81, and CD9 (Fig. 1G). CD63 was detected at approximately 53 kDa, CD81 at 26 kDa, and CD9 at 25 kDa, consistent with their expected molecular weights.

### NCSC-sEVs Interact with Glioblastoma Cells

To examine their ability to interact with U251 glioblastoma cells, sEVs were fluorescently labelled with PKH67, while U251 glioblastoma cells were pre-labelled with CellTracker Red.

Confocal microscopy analysis revealed uptake of the PKH67-labelled sEVs and binding to glioblastoma cell membranes (Fig. 1H). Merged fluorescence images demonstrated clear co-localisation of sEVs with the glioblastoma cells. The sEVs appeared as distinct punctate structures distributed throughout the cytoplasm, indicating internalisation as well as membrane binding.

### NCSC-sEVs and Secretomes inhibit TNF-α-driven Increase in Cell Viability

**Fig. 2:**
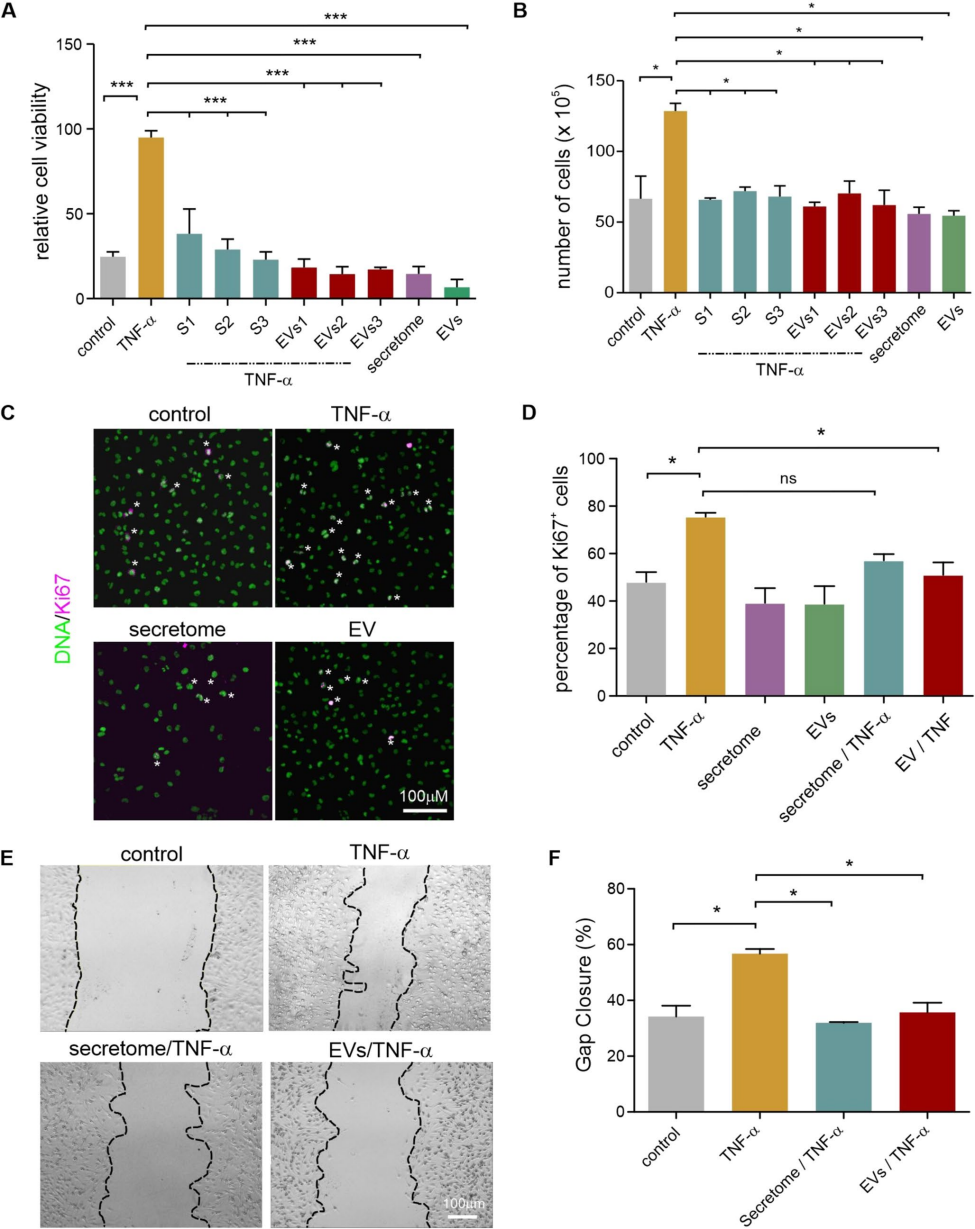
NCSC secretomes and sEVs prevent TNF-α-driven increase in glioblastoma cell viability, proliferation, and migration.** A** XTT viability assay indicated TNF-α-induced increase of U251 cell viability and its inhibition by NCSC secretomes and sEVs. **B**. Automated cell counting demonstrating TNF-α-induced increase in cell numbers and prevention by secretome and sEV co-treatment. **C** Representative immunocytochemical images of Ki67 staining (green) in U251 cells under different treatment conditions. Examples of marker-positive cells are highlighted with asterisks (**D**) Quantification of Ki67-positive cells showed TNF-α-induced proliferation and reduction to control levels by sEV co-treatment. **E** Representative images from wound healing migration assay. **F** Quantification of wound closure demonstrated TNF-α-induced increase of migration (~ 55% vs. 35% control) and its reduction to control levels by secretome/sEV co-treatment. Data represent mean ± SEM from three independent experiments. *p < 0.05, **p < 0.01, ***p < 0.001

Assessment of cell viability revealed that TNF-α significantly enhanced U251 cell viability compared to untreated controls (Fig. 2A). This effect was inhibited by NCSC secretomes and sEVs. Statistical analysis revealed significant differences (*p* < 0.001) between TNF-α-treated cells and those co-treated with secretomes or EVs.

### NCSC-sEVs and Secretomes Prevent TNF-α-induced Increase in Cell Proliferation

To investigate the potential of NCSC-sEVs and secretomes against inflammation-driven glioblastoma progression, we examined their effects on TNF-α-stimulated U251 cell proliferation. Cell counting demonstrated that TNF-α significantly increased cell numbers compared to control conditions (Fig. 2B). Co-treatment with secretomes or sEVs and TNF-α prevented the TNF-α-induced increase in proliferation. To validate the results of the total cell number counting, cells were stained for Ki67. In control cultures, Ki67-positive nuclei were abundant, indicating active cell proliferation (Fig. 2C), with a significant increase in Ki67-positive cells in response to TNF-α. Pre-incubation and co-treatment with sEVs reduced the proportion of Ki67-positive nuclei compared to TNF-α.

### NCSC-secretomes and sEVs Inhibit Glioblastoma Cell Migration

Wound healing assays demonstrated the anti-migratory effects of NCSC products (Fig. 2E). TNF-α treatment significantly enhanced gap closure compared to control conditions. However, co-treatment with either secretomes or sEVs reduced gap closure to levels comparable to controls.

### NCSC Secretomes Prevent TNF-α-induced Nuclear Translocation of NF-κB

**Fig. 3:**
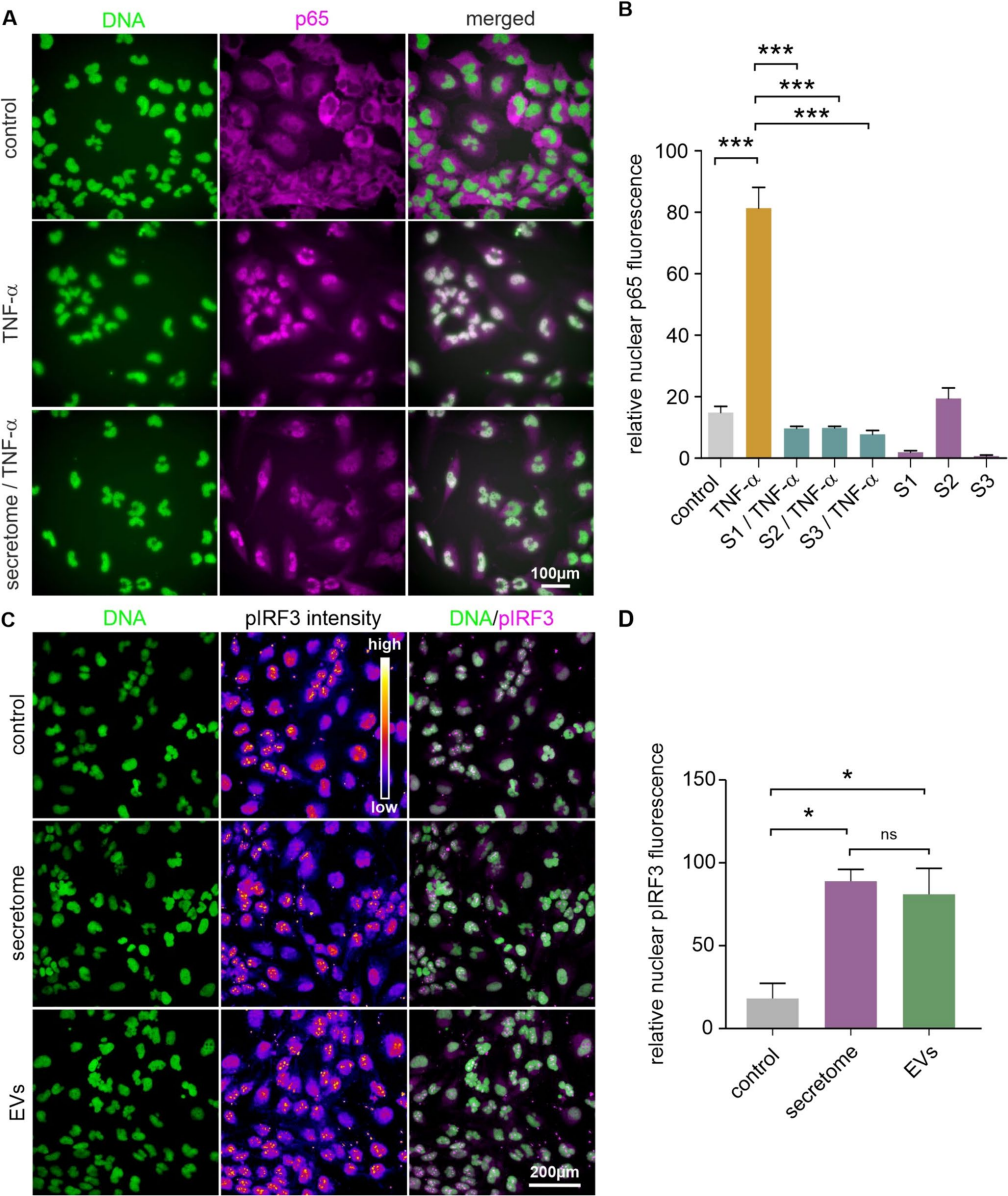
NCSC secretomes prevent TNF-α-induced NF-κB nuclear translocation and induce nuclear translocation of phosphorylated IRF3. **A** Representative spinning disc confocal microscopy images showing subcellular localisation of NF-κB p65 subunit (magenta) in U251 cells. Control cells showed predominantly cytoplasmic localisation, TNF-α treatment induced nuclear translocation, while secretome co-treatment prevented nuclear accumulation of p65. Cell nuclei are counterstained with DAPI (green). Scale bars represent 100 μm. **B** Quantitative analysis of nuclear p65 fluorescence intensity indicated a ~ 5-fold increase after TNF-α treatment and a significant reduction by secretome co-treatment across all donors (*p* < 0.001 for all comparisons with TNF-α alone). Data represent mean ± SEM from three independent experiments. ***p < 0.001. **C** Representative confocal microscopy images showing subcellular localisation of phosphorylated IRF3 (pIRF3) intensity (displayed as heat map from low to high) in U251 cells. Cell nuclei are stained with DAPI (green). Images demonstrated enhanced nuclear pIRF3 staining in secretome and sEV-treated cells compared to controls. Scale bar: 200 μm. **D** Quantitative analysis of nuclear pIRF3 fluorescence showing a significant increase after exposure to both secretome and sEVs compared to control conditions (*p < 0.05). No significant difference was observed between secretome and sEV treatments (ns). Data represent mean ± SEM from three independent experiments. *p < 0.05

We examined NF-κB activation using ICC for the p65 subunit and machine learning-driven analysis of its subcellular localisation. In control cells, p65 exhibited predominantly cytoplasmic localisation (Fig. 3A).

TNF-α treatment induced nuclear translocation, as evidenced by co-localisation of the p65 signal with DAPI staining. Co-treatment with NCSC secretomes reduced the levels of TNF-α-induced nuclear translocation. Quantitative analysis confirmed these observations (Fig. 3B). TNF-α treatment significantly increased nuclear p65 fluorescence compared to controls. This was prevented by co-treatment with secretomes, with all treatments reducing the levels of nuclear p65 to near-control values. Additional p65 translocation analyses have been performed in U373 and U87 glioblastoma cells (Figure S2).

Similar to U251 cells, control cells displayed baseline cytoplasmic p65 distribution, whilst TNF-α treatment induced nuclear accumulation. Co-treatment with NCSC secretomes prevented nuclear translocation of p65, maintaining the cytoplasmic distribution pattern.

### Secretomes and sEVs Induce Nuclear Translocation of IRF3

We examined the effects of NCSC secretomes and sEVs on activation of IRF3 using ICC for total IRF3 (Figure S3) and phosphorylated IRF3 (pIRF3, Fig. 3C), followed by analysis of the subcellular localisation.

Images revealed nuclear staining patterns in treated cells, consistent with IRF3 translocation and transcriptional activation. Analysis revealed that NCSC secretomes and sEVs significantly enhanced nuclear translocation of total IRF3 and pIRF3 compared to control conditions (Fig. 3D, Figure S3B).

###  NCSC Secretomes and EVs Prevent NF-κB Activation and Robustly Activate IRF3

**Fig. 4:**
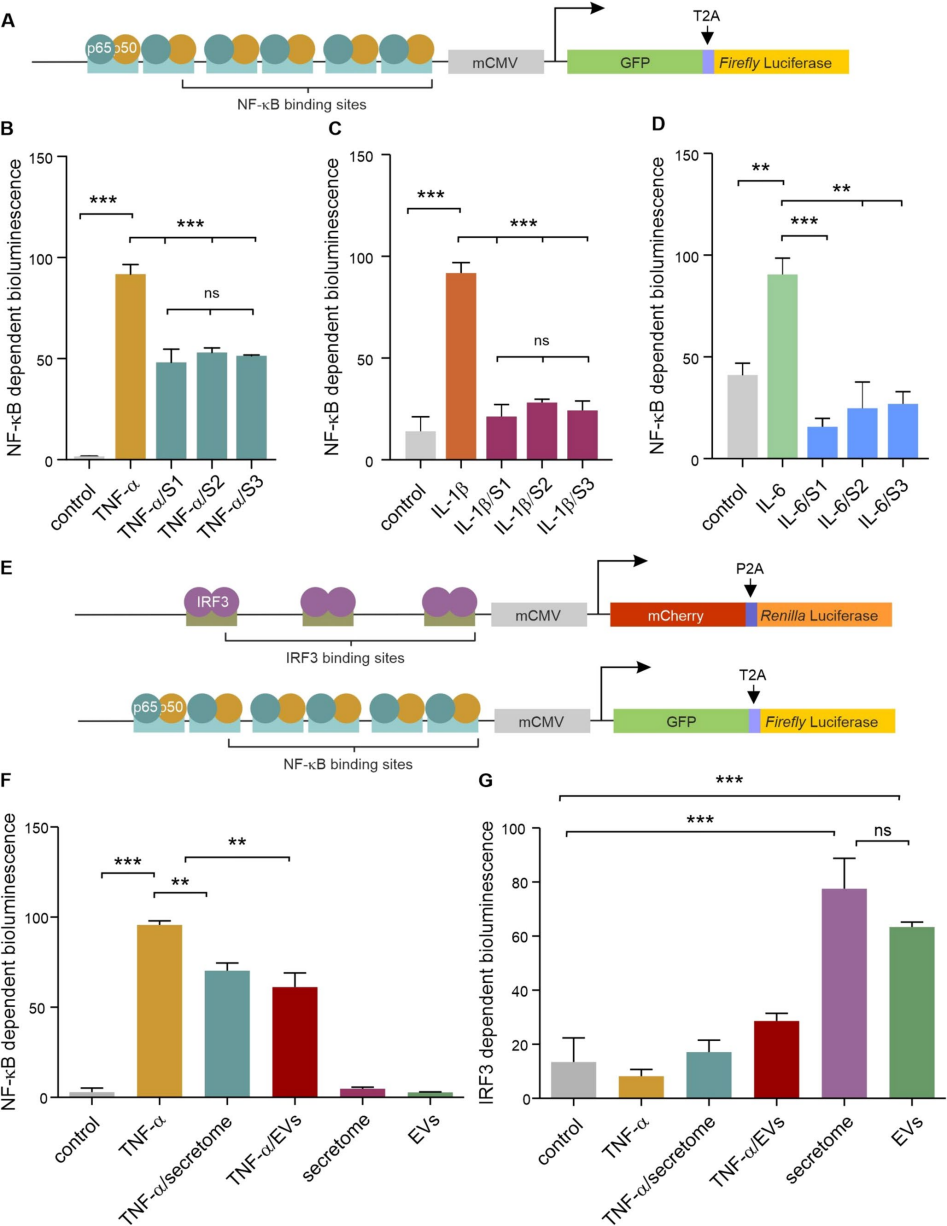
Dual NF-κB gene reporter assays and quadruple NF-κB/IRF3 gene reporter assays confirm inflammatory rebalancing by NCSC secretomes and sEVs. A. Schematic representation of the dual NF-κB reporter system. **B-D.** The activity of the NF-κB was robustly activated by the pro-inflammatory factors TNF-α, IL-1β, and IL-6. This pro-inflammatory response was reduced after co-exposure to the respective pro-inflammatory factors and NCSC secretomes or EVs. **E.** Schematic of quadruple NF-κB/IRF3 reporter system allowing simultaneous pathway monitoring. **F-G**. Quadruple reporter analysis demonstrated TNF-α-mediated activation of NF-κB without IRF3 response, with a significant reduction after co-exposure to secretome (donor 3)/sEVs. Both secretomes and sEVs substantially increased IRF3 activity compared to control levels. Data represent mean ± SEM from three independent experiments. *p < 0.05, **p < 0.01, ***p < 0.001

To quantitatively assess the anti-inflammatory potential of NCSC products, we used a previously established U251 NF-κB reporter cell line (Fig. 4A) [39]. Using this reporter system, we demonstrated that TNF-α treatment resulted in NF-κB activation (Fig. 4B).

Co-treatment of cells with NCSC secretomes or sEVs significantly attenuated TNF-α-induced response. IL-1β-treatment induced substantial NF-κB activation, which was significantly reduced by both secretome and sEV co-treatment (Fig. 4C). Similarly, IL-6-induced NF-κB activity was effectively suppressed by both NCSC products (Fig. 4D).

To simultaneously monitor NF-κB and IRF3 activity, we developed a quadruple reporter system by transducing the NF-κB reporter cells with a lentivirus containing an IRF3-responsive construct (Fig. 4E). Using this quadruple reporter system, we observed that TNF-α treatment activated NF-κB without IRF3 activation (Fig. 4F). NCSC secretomes and sEVs significantly inhibited TNF-α-induced NF-κB activity. Both secretomes and sEVs substantially enhanced IRF3 activity (Fig. 4G). This was particularly pronounced, with both treatments increasing IRF3 activity to levels significantly higher than the controls. There was no significant difference between secretomes and sEVs for IRF3 activation.

### NCSC Secretomes and sEVs Reprogram the Secretory Profiles of Glioblastoma Cells towards an Anti-inflammatory and Regulatory State

**Fig. 5:**
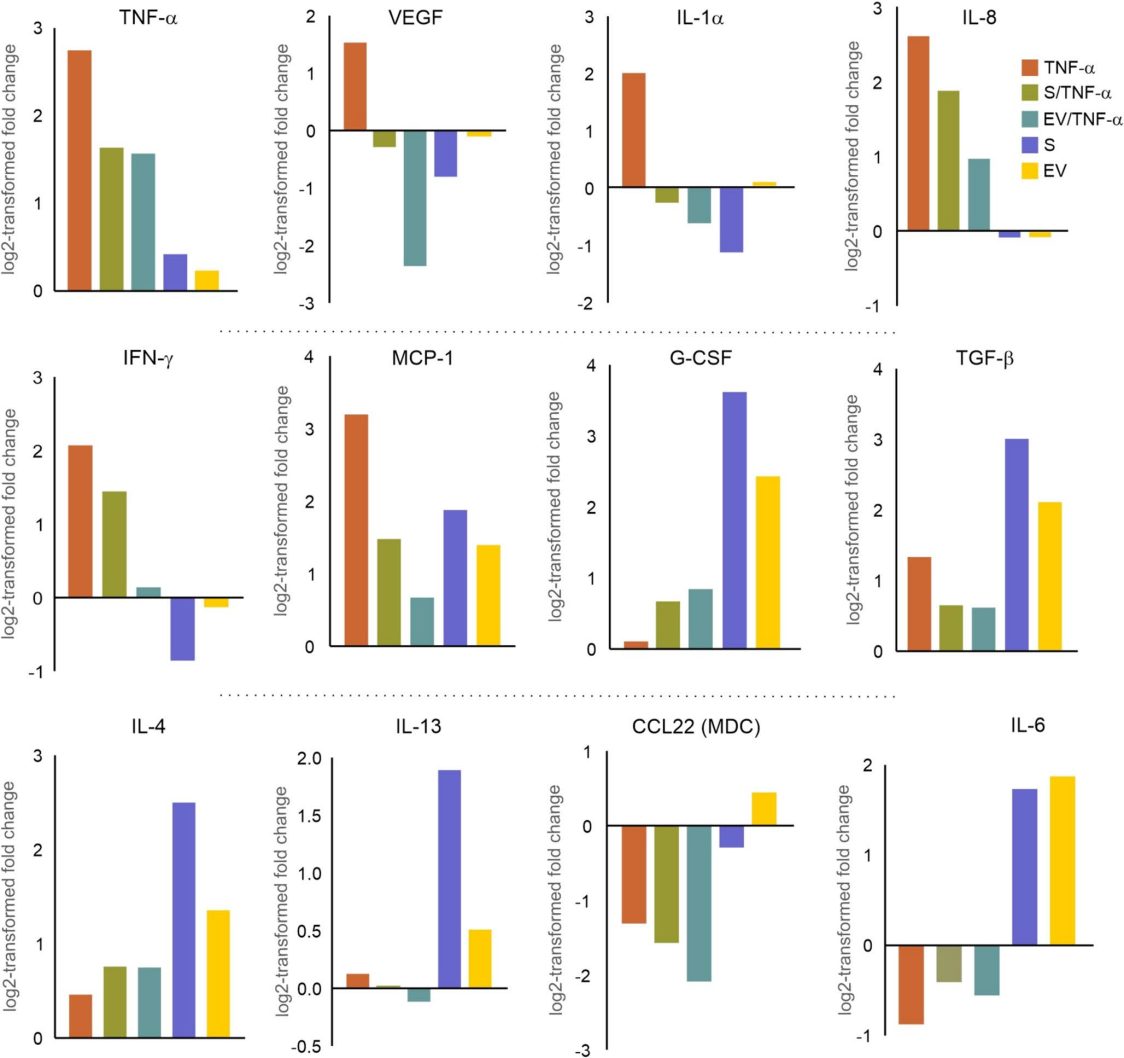
NCSC secretomes and sEVs reprogram glioblastoma cytokine secretion profiles. Semi-quantitative cytokine array analysis of conditioned media from U251 cells showing log2-transformed fold changes relative to control. TNF-α treatment substantially increased pro-inflammatory cytokines (TNF-α, IFN-γ, IL-1α, IL-8, MCP-1, and VEGF. Co-treatment with secretome (donor 3) or sEVs reduced the levels of pro-inflammatory factors while increasing regulatory factors (TGF-β, G-CSF). Data represent the mean from three independent experiments

To assess the cytokine secretory profile of glioblastoma cells, we used a semi-quantitative human cytokine antibody array. TNF-α treatment substantially increased the secretion of multiple pro-inflammatory cytokines (Fig. 5).

In addition to TNF-α itself, other upregulated cytokines included IFN-γ, IL-1α, and IL-8. Co-treatment with NCSC products substantially attenuated the TNF-α-induced upregulation of TNF-α, IFN-γ, IL-1α, and IL-8 secretion. The analysis also revealed complex modulation of growth factors and angiogenic factors. TNF-α treatment increased VEGF secretion, which was suppressed by NCSC products. While TNF-α alone had minimal effects on TGF-β1 levels, both secretomes and sEVs substantially increased TGF-β1 secretion. TNF-α treatment upregulated several chemokines, including MCP-1, which was significantly reduced by NCSC product co-treatment. In contrast, anti-inflammatory and regulatory cytokines showed enhanced expression following NCSC product treatment. G-CSF levels were increased by sEVs alone, suggesting immunomodulatory effects independent of TNF-α stimulation. While secretomes and sEVs showed similar anti-inflammatory profiles, IL-4 and IL-13 showed more pronounced increases after sEV treatment. Additionally, IL-6 levels were differentially modulated, with sEVs showing greater suppression of TNF-α-induced IL-6 upregulation.

### NCSC Secretomes and their sEVs Inhibit Tumourigenic Properties of Glioblastoma

**Fig. 6:**
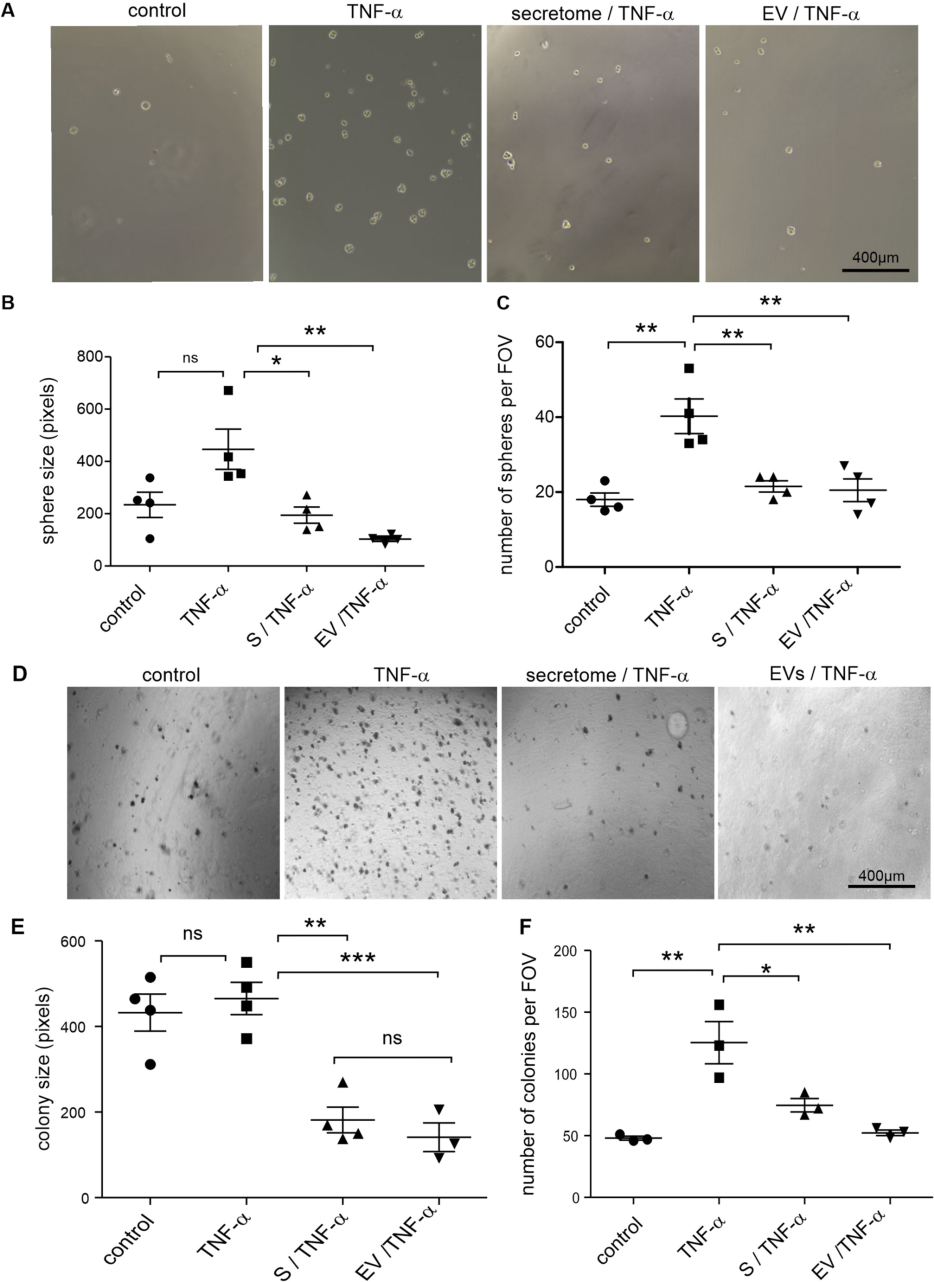
NCSC secretomes and sEVs inhibit tumourigenic properties of glioblastoma cells.** A** Representative images of tumourisphere formation under different experimental conditions. Scale bars represent 400 μm. **B** Quantification of tumourisphere size indicated a trend towards a TNF-α-induced increase in size and a significant reduction by secretome (donor 3)/sEV co-treatment compared to the TNF group. **C** Quantitative analysis of the tumourisphere number showed a significant increase in response to TNF-α, which was prevented by co-exposure to secretome and sEVs. Data represent mean ± SEM from three independent experiments. *p < 0.05, **p < 0.01, ***p < 0.001. **D** Representative images of colony formation in soft agar. Scale bars represent 400 μm. E-F. Quantitative analysis of colony size and number per field of view (FOV). TNF-α increased colony number without affecting size, while secretome/sEV co-treatment significantly reduced both parameters compared to TNF-α alone and controls. Data represent mean ± SEM from three independent experiments. *p < 0.05, **p < 0.01, ***p < 0.001

Tumourigenicity was assessed using the tumourisphere formation assay. TNF-α treatment tended towards an increase in tumourisphere size (Fig. 6A), although this did not reach statistical significance.

However, exposure to NCSC products resulted in a significant reduction in sphere sizes compared to TNF-α alone. In contrast, the number of tumourispheres per FOV (Fig. 6C) was strongly elevated following TNF-α stimulation and was reduced by NCSC product co-treatment.

Effects of NCSC secretomes and sEVs on the anchorage-independent growth were evaluated using soft agar assays. Representative images (Fig. 6D) show the morphological appearance of colonies under different treatment conditions.

Quantitative analysis (Fig. 6E) showed that TNF-α treatment alone did not significantly alter the size of the colonies. However, co-treatment with NCSC products led to a significant reduction in colony size compared to the TNF-α-alone and control groups. The number of colonies per FOV was significantly increased following TNF-α exposure (Fig. 6F). This was significantly reduced by co-treatment with secretome or sEVs.

### Secretomes and sEVs Enhance the Anti-tumour effects of TMZ in Glioblastoma Cells

TMZ treatment resulted in a significant reduction in cell viability compared to the control (Figure S4).

**Fig. 7:**
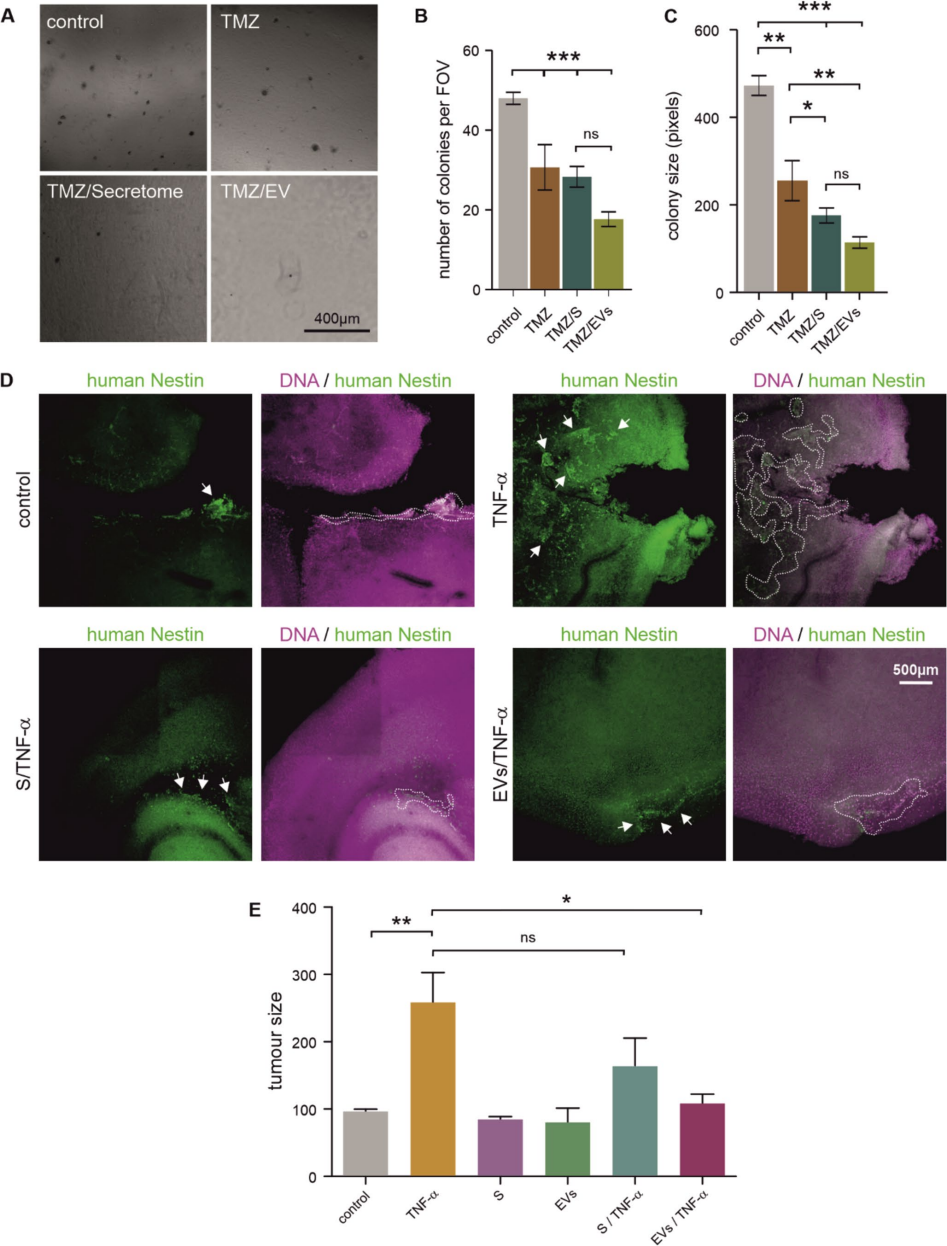
NCSC products increase chemosensitivity to TMZ and reduce tumour growth in organotypic brain slices. **A**. Representative images of soft agar colonies under TMZ ± secretome/sEV treatment. Bar represents 400 μm. **B-C.** Quantification of colony number and size revealed a significant reduction of both parameters in response to TMZ. While the number of colonies was not affected by co-exposure to TMZ and secretomes of sEVs, both combinations resulted in a significant decrease in colony size compared to TMZ alone. **D**. Representative confocal images of organotypic mouse hippocampal slices with transplanted U251 cells (identified by human-specific nestin staining (green)) under different treatment conditions. Nuclei were counterstained with DAPI (magenta). Bar corresponds to 500 μm. Quantification of tumour area showing TNF-α-induced growth and effective prevention by secretome/sEV co-treatment. Secretomes/sEVs alone maintain tumour sizes comparable to controls. Clusters of human glioma cells are highlighted with arrows in the human nestin channel, and traced in the respective merged channels. Data represent mean ± SEM from three independent experiments. *p < 0.05, **p < 0.01, ***p < 0.001

Both NCSC products significantly reduced cell viability. Co-treatment with secretomes combined with TMZ did not show additional significant reduction in viability compared with TMZ alone. However, combination treatments with sEVs from donors 1 and 3 resulted in a further decrease compared to TMZ monotherapy. The effect of the treatments on anchorage-independent growth was assessed with the soft agar assay (Fig. 7A-C). Representative images of the colonies formed under each treatment are shown in Fig. 7A. Quantitative analysis revealed that TMZ treatment significantly reduced the number of colonies per FOV compared to controls (Fig. 7B, *p* < 0.001). Colony size analysis showed that TMZ treatment alone resulted in a significant reduction in colony size compared to control (Fig. 7C). The combination of secretomes and TMZ caused an even greater reduction in colony size compared with TMZ alone, while the combination of TMZ and sEVs produced the smallest colonies overall.

### NCSC Secretomes and EVs Reduce Tumour Size in an Organotypic Mouse Brain Slice Model

U251 glioblastoma cells were transplanted onto hippocampal slices, and tumour growth was assessed after seven days in culture. Quantification of tumour area (Fig. 7D) showed that TNF-α significantly increased tumour size compared to control. In contrast, slices treated with NCSC products alone exhibited tumour sizes comparable to the controls.

Co-treatment with NCSC products effectively prevented TNF-α-induced tumour enlargement. Tumour sizes in secretome/TNF-α and EV/TNF-α groups were significantly reduced compared to TNF-α alone, and not significantly different from control, secretome, or EV treatment alone.

### NCSC Secretomes and sEVs Preserve Organotypic Brain Slice Viability

To ensure that the observed anti-tumour effects were not due to general cytotoxicity, we assessed tissue viability using LDH release assays. Analysis on day 1 post-treatment revealed that none of the experimental conditions caused significant cytotoxicity compared to untreated controls (Figure S5A).

Extended viability assessment on day 4 post-treatment confirmed the absence of delayed cytotoxic effects (Figure S5B).

## Discussion

This study is the first comprehensive demonstration of dual-pathway inflammatory rebalancing by NCSC secretomes and sEVs in glioblastoma, introducing a novel therapeutic paradigm that simultaneously suppresses tumour-promoting NF-κB signalling whilst enhancing anti-tumour IRF3 pathways. We present compelling evidence for the therapeutic potential of NCSC-derived products as adjuvants that address multiple resistance mechanisms underlying the therapy resistance of glioblastoma.

The prominent role of NF-κB in glioblastoma pathogenesis has been extensively documented, with constitutive activation driving proliferation, invasion, angiogenesis, and resistance to both radiotherapy and chemotherapy (reviewed in [45]). Our results confirm and extend these findings, demonstrating that TNF-α-induced NF-κB activation significantly enhances glioblastoma cell viability, proliferation, and migration.

NCSCs from different sources promote regeneration in multiple pre-clinical disease models [17, 20, 46, 47]. Notably, their regenerative effects are at least partly mediated by paracrine effects rather than solely by differentiation and tissue integration [17, 47]. Indeed, NCSC secretomes and EVs exert their therapeutic effects primarily through anti-inflammatory and immunomodulatory mechanisms, including NF-κB pathway suppression, cytokine profile rebalancing, and immune cell phenotype switching [48–50]. In our study, we demonstrated that NCSC secretomes and sEVs potently suppress TNF-α-, IL-1β-, and IL-6-induced NF-κB activation, as evidenced by a profound reduction in p65 nuclear translocation. Reporter assays confirmed these findings, showing a clinically meaningful level of NF-κB suppression, particularly as direct pharmacological NF-κB inhibition has proven toxic in clinical trials [51].

Research has highlighted key roles for NF-κB in maintaining glioma stem-like cell properties, metabolic adaptation, and regulating pro-tumorigenic cytokine production within the tumour microenvironment (reviewed in [52]). Our cytokine array data demonstrate that NCSC products effectively reprogram this inflammatory milieu, suppressing multiple pro-inflammatory mediators (TNF-α, IL-1α, IL-8, IFN-γ, MCP-1) while enhancing regulatory factors such as TGF-β1. This is consistent with evidence that NF-κB modulation can shift glioblastoma transcriptional subtypes from mesenchymal towards less aggressive pro-neural phenotypes [8, 53].

Simultaneous activation of IRF3 represents a novel aspect of our findings. While IRF3 is recognised for its anti-viral functions, it is also a negative regulator of glioma invasion and proliferation [11, 37]. The increased nuclear translocation of IRF3 and enhanced transcriptional activity by NCSC products suggests activation of anti-tumour immune surveillance mechanisms. The significance here, is it that IRF3 can inhibit NF-κB p65 nuclear translocation, establishing a potential molecular basis for the dual-pathway modulation [37]. This dual regulation of tumour-promoting pathways whilst enhancing tumour-suppressive signalling represents a novel approach that may overcome compensatory mechanisms often observed with single-pathway inhibition strategies [54, 55].

Glioma stem cells (GSCs) are inherently chemo- and radioresistant due to enhanced DNA repair mechanisms, anti-apoptotic protein expression, and metabolic adaptability [56, 57]. We demonstrated significant reductions in tumourisphere formation and colony growth in response to NCSC secretomes and sEVs, indicating that NCSC products can effectively target glioblastoma stem-like cell properties. In this context, the NF-κB pathway maintains GSC stemness and transcriptional subtype plasticity [58], suggesting that its modulation by NCSC products may reduce the stem cell population that is driving therapeutic resistance.

The current standard-of-care for GBM patients is surgical resection of the tumour followed by chemotherapy with TMZ. However, TMZ resistance remains a major clinical challenge, with multiple mechanisms including MGMT-mediated DNA repair, enhanced survival signalling, and EV-mediated resistance transfer [59]. A recent meta-analysis provided clinical evidence that combination therapies that combine TMZ with drugs such as bevacizumab can improve clinical outcomes [60]. Our results demonstrated that NCSC products can enhance the cytotoxic effects of TMZ. While NCSC secretomes or sEVs alone did not reach the cytotoxic potency of TMZ, they significantly enhanced its anti-tumour effects, yielding additive or synergistic effects. The observed effects, particularly the significant reduction in colony size in soft agar assays when combining TMZ with sEVs, suggest that NCSC products may overcome some of the resistance mechanisms.

The hippocampal slice model provided critical validation of our findings in a tissue-relevant context that preserves the complex cellular interactions of the brain microenvironment. Consistent with previous studies demonstrating the role of TNF-α in promoting glioblastoma progression within brain tissue [61, 62], TNF-α treatment significantly increased tumour area compared to controls, confirming the pro-tumorigenic effects of inflammatory signalling in the brain environment. The ability of NCSC-derived products to suppress tumour growth while preserving normal brain tissue integrity strongly supports their translational potential for glioblastoma therapy. We elected to use an organotypic hippocampal brain slice model because it preserves native brain cytoarchitecture, neuronal networks, and key microenvironmental components while remaining experimentally tractable. This ex vivo system enables controlled co-culture of human glioblastoma cells with intact murine brain tissue, high-content imaging, and quantitative analysis of tumour area and slice viability without the systemic confounders, inter-animal variability, and low throughput inherent to orthotopic in vivo xenografts. In addition, the slice model is particularly suited to parallel testing of multiple NCSC-derived products and temozolomide combinations under defined inflammatory conditions and aligns with the 3Rs by substantially reducing animal use and avoiding long-term procedures.

Adult NCSCs have demonstrated safety in preclinical studies, with no evidence of tumourigenicity [63]. However, studies have highlighted the importance of careful characterisation of stem cell derivatives, as even differentiated cells may retain some oncogenic properties [64–66]. Thus, the safety profile of NCSC-derived products represents a significant advantage over whole-cell therapies. Moreover, EVs can be standardised under GMP conditions, engineered for targeted delivery, and exhibit reduced immunogenicity compared to stem cells [67, 68]. Consistent activity across three independent donors suggests robust manufacturing potential. Advances in EV engineering and targeted delivery systems provide opportunities for further optimising NCSC-derived therapeutics. The ability to modify EV cargo could enhance tumour targeting while minimising off-target effects [30, 69, 70].

While our comprehensive validation provides strong preclinical evidence, the heterogeneity between different glioblastoma subtypes suggests that treatment responses may vary across patients. Thus, future studies should investigate the differential sensitivity of pro-neural versus mesenchymal subtypes to NCSC-derived products, potentially enabling personalised treatment approaches. Moreover, future in vivo validation in orthotopic glioblastoma models will provide insights regarding blood-brain barrier penetration, biodistribution, and therapeutic efficacy in the complex tumour microenvironment.

The dual-pathway modulation by NCSC products suggests several clinical implementation strategies. The enhancement of TMZ efficacy supports development as an adjuvant therapy alongside standard treatment. The anti-inflammatory properties may also synergise with emerging immunotherapeutic approaches to overcome the immunosuppressive microenvironment that limits checkpoint inhibitor efficacy [71].

## Conclusion

The study demonstrates for the first time that NCSC-derived secretomes and sEVs exert potent anti-glioblastoma effects by simultaneously suppressing pro-tumour NF-κB signalling and activating tumour-suppressive IRF3 pathways. This dual mechanism reduced tumour cell viability, proliferation, and migration, while reprogramming the tumour cytokine milieu towards anti-inflammatory profiles. Importantly, NCSC secretomes and sEVs enhanced the chemotherapeutic efficacy of TMZ and prevented inflammation-driven tumour growth in ex vivo brain slice models, establishing a novel, therapeutically significant paradigm of inflammatory rebalancing with potential for clinical translation. Our GMP-compatible cell-free approach overcomes the safety and scalability limitations of cellular therapies whilst offering superior therapeutic targeting compared to single-agent approaches that have consistently failed in clinical trials.

## Supplementary Information

Below is the link to the electronic supplementary material.

Supplementary figure 8 (PNG 1.32 MB)

NCSC characterisation. Immunocytochemical characterisation of human oral mucosa NCSCs. Confocal microscopy revealed robust expression of the neural crest stem cell marker nestin with a characteristic filamentous cytoplasmic staining pattern. Cells demonstrate typical elongated morphology consistent with NCSCs. Cell nuclei are counterstained with DAPI (blue). Scale bar represents 200 μm. High Resolution Image (TIF 2.77 MB)

Supplementary figure 9 (PNG 1.50 MB)

**NCSC secretomes prevent NF-κB activation across multiple glioblastoma cell lines. (A)** Representative confocal microscopy images showing NF-κB p65 nuclear translocation in U373 cells. Similar to U251 cells, control conditions showed cytoplasmic distribution, TNF-α treatment induced pronounced nuclear accumulation, and NCSC secretome co-treatment prevented nuclear translocation of p65, maintaining its cytoplasmic distribution. Cell nuclei were counterstained with DAPI (green). Scale bar represents 100 μm. **(B)** Quantitative analysis demonstrated nuclear translocation of p65 in response to TNF-α, which was prevented by co-exposure to secretome or sEVs. *p < 0.05, **p < 0.01. **(C)** Confocal images showing subcellular localisation of p65 (magenta) in U87 cells. Nuclei were counterstained with DAPI (green). RFU: relative fluorescence unit). Scale bar: 100 μm. **(D)** Quantitative analysis demonstrated a TNF-α-induced increase in nuclear translocation of p65, which was reduced after co-exposure to secretomes and sEVs. *p < 0.05, **p < 0.01. High Resolution Image (TIF 4.05 MB)

Supplementary figure 10 (PNG 529 KB)

**NCSC secretomes and sEVs induce IRF3 nuclear translocation. (A)** Representative spinning disc confocal microscopy images showing subcellular localisation of total IRF3 (magenta) in U251 cells treated with control, secretome, or sEVs. Nuclear translocation was evident in treated cells. Cell nuclei are counterstained with DAPI (green). Scale bar represents 100 μm. **(B)** Quantitative analysis showing significant enhancement of nuclear IRF3 translocation by both secretomes and sEVs compared to control conditions. Data represent mean ± SEM from three independent experiments. *p < 0.05, **p < 0.01. High Resolution Image (TIF 1.69 MB)

Supplementary figure 11 (PNG 30.3 KB)

**Dual NF-κB gene reporter assays and quadruple NF-κB/IRF3 gene reporter assays confirm inflammatory rebalancing by NCSC secretomes and sEVs. A.** Schematic representation of the dual NF-κB reporter system. **B-D.** The activity of the NF-κB was robustly activated by the pro-inflammatory factors TNF-α, IL-1β, and IL-6. This pro-inflammatory response was reduced after co-exposure to the respective pro-inflammatory factors and NCSC secretomes or EVs. **E.** Schematic of quadruple NF-κB/IRF3 reporter system allowing simultaneous pathway monitoring. **F-G**. Quadruple reporter analysis demonstrated TNF-α-mediated activation of NF-κB without IRF3 response, with a significant reduction after co-exposure to secretome (donor 3)/sEVs. Both secretomes and sEVs substantially increased IRF3 activity compared to control levels. Data represent mean ± SEM from three independent experiments. *p < 0.05, **p < 0.01, ***p < 0.001. High Resolution Image (TIF 161 KB)

Supplementary figure 12 (PNG 92.7 KB)

**NCSC products increase chemosensitivity to TMZ.** XTT viability assay demonstrated TMZ-induced cytotoxicity and increased chemosensitivity with sEV from donors 1 and 3. Both secretomes and sEVs alone significantly reduce viability compared to controls. High Resolution Image (TIF 262 KB)

## Data Availability

All relevant data supporting the findings of this study are available within the paper, its supplementary information and from the corresponding authors upon reasonable request.
