# Supplementary figures and images for: Neural Crest-Derived Stem Cell Secretomes and Extracellular Vesicles Disrupt Glioblastoma through Dual-Pathway Inflammatory Rebalancing
Source: Stem Cell Rev Rep. 2026 Apr 28;22(5):2528–48. doi: 10.1007/s12015-026-11133-5 (PMC13241452; doi:10.1007/s12015-026-11133-5)

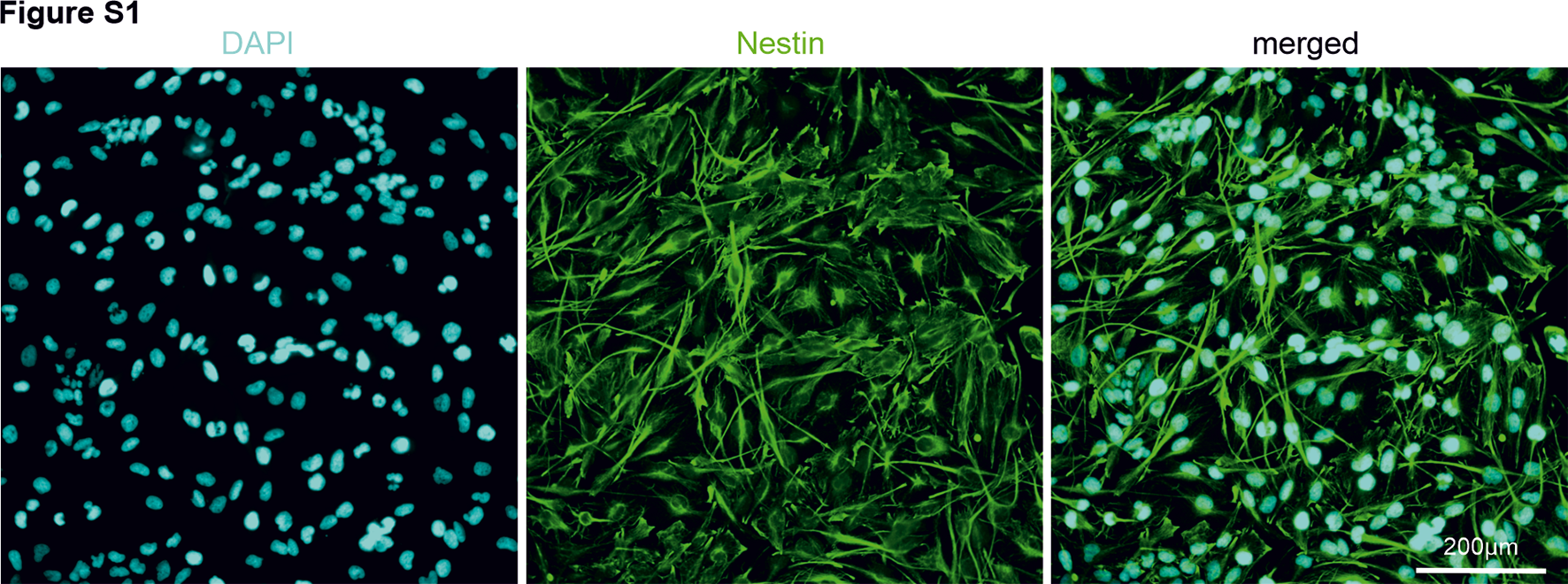

Supplement: Supplementary file 1 — (PNG 1.32 MB) [file 12015_2026_11133_Fig8_ESM.png]

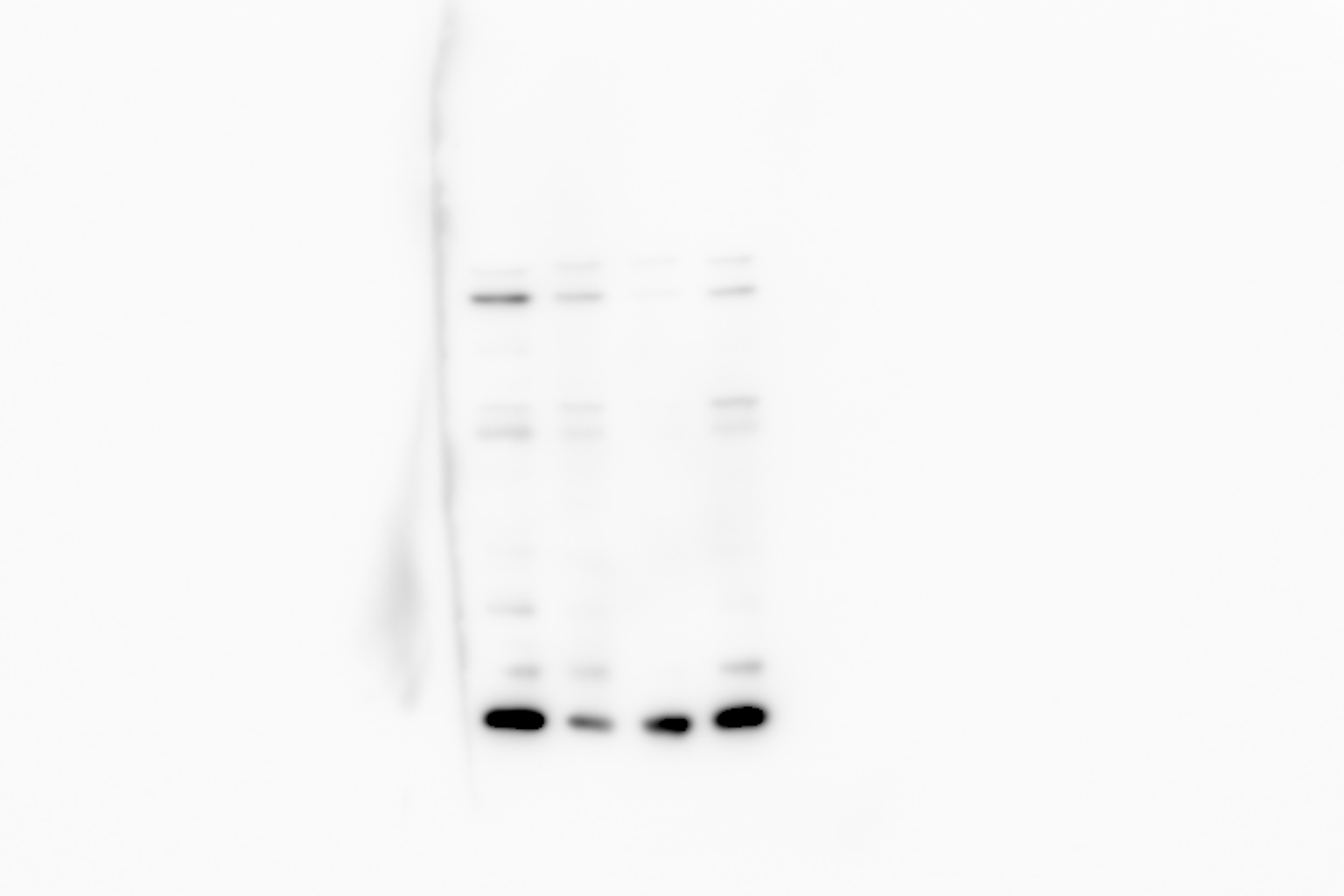

Supplement: Supplementary file 2 — NCSC characterisation. Immunocytochemical characterisation of human oral mucosa NCSCs. Confocal microscopy revealed robust expression of the neural crest stem cell marker nestin with a characteristic filamentous cytoplasmic staining pattern. Cells demonstrate typical elongated morphology consistent with NCSCs. Cell nuclei are counterstained with DAPI (blue). Scale bar represents 200 μm. High Resolution Image (TIF 2.77 MB) [file 12015_2026_11133_MOESM1_ESM.tif]

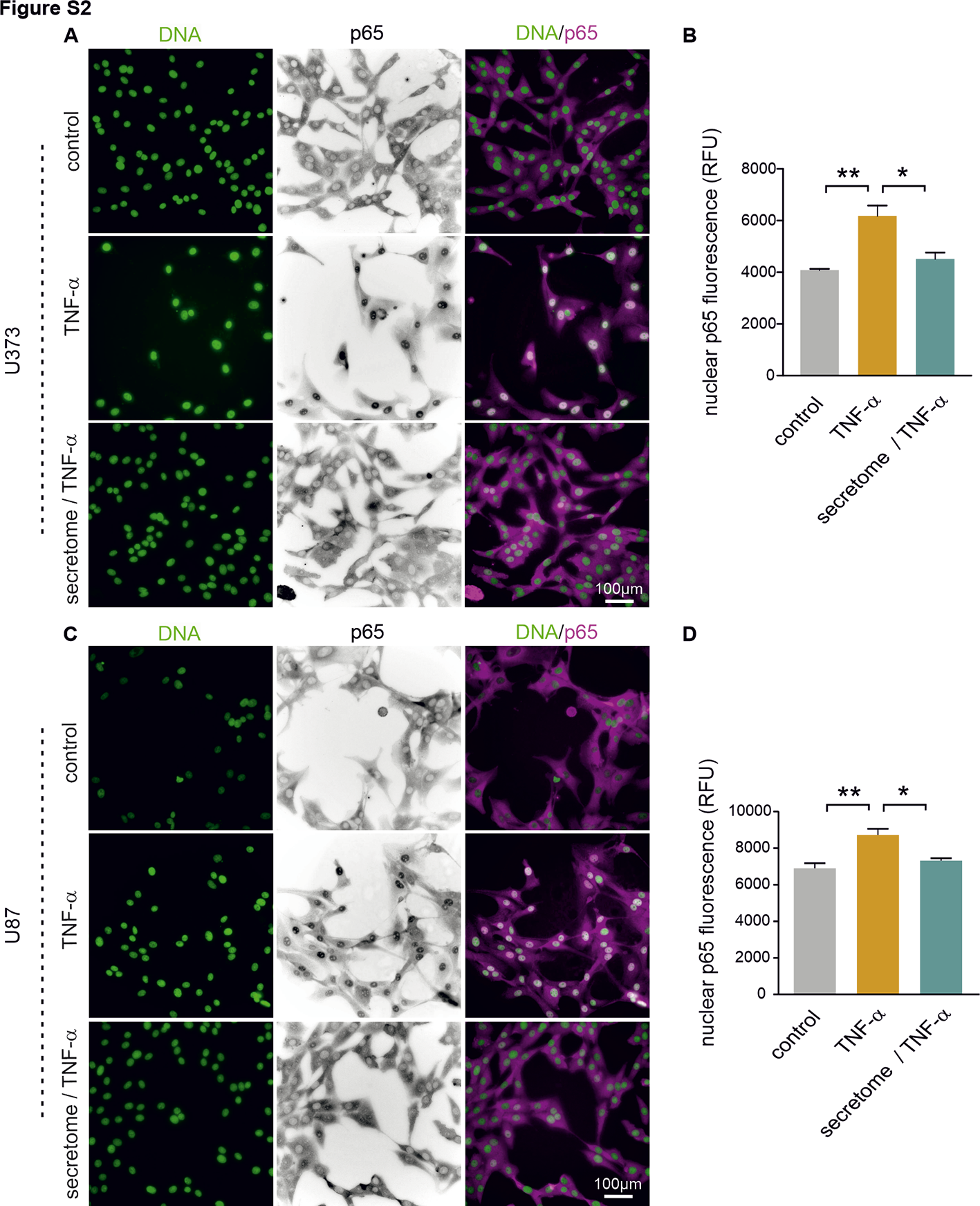

Supplement: Supplementary file 3 — (PNG 1.50 MB) [file 12015_2026_11133_Fig9_ESM.png]

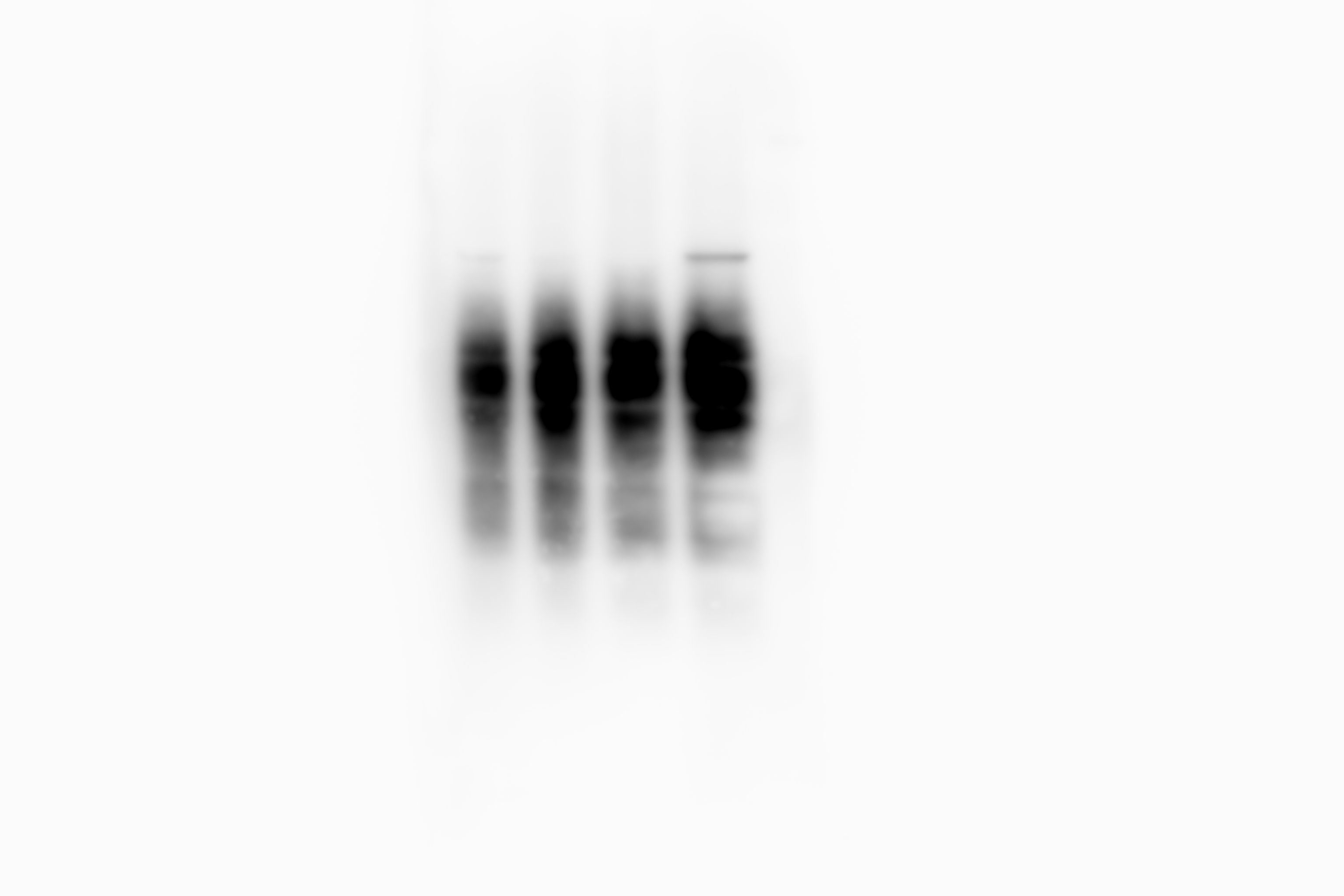

Supplement: Supplementary file 4 — NCSC secretomes prevent NF-κB activation across multiple glioblastoma cell lines. (A) Representative confocal microscopy images showing NF-κB p65 nuclear translocation in U373 cells. Similar to U251 cells, control conditions showed cytoplasmic distribution, TNF-α treatment induced pronounced nuclear accumulation, and NCSC secretome co-treatment prevented nuclear translocation of p65, maintaining its cytoplasmic distribution. Cell nuclei were counterstained with DAPI (green). Scale bar represents 100 μm. (B) Quantitative analysis demonstrated nuclear translocation of p65 in response to TNF-α, which was prevented by co-exposure to secretome or sEVs. *p < 0.05, **p < 0.01. (C) Confocal images showing subcellular localisation of p65 (magenta) in U87 cells. Nuclei were counterstained with DAPI (green). RFU: relative fluorescence unit). Scale bar: 100 μm. (D) Quantitative analysis demonstrated a TNF-α-induced increase in nuclear translocation of p65, which was reduced after co-exposure to secretomes and sEVs. *p < 0.05, **p < 0.01. High Resolution Image (TIF 4.05 MB) [file 12015_2026_11133_MOESM2_ESM.tif]

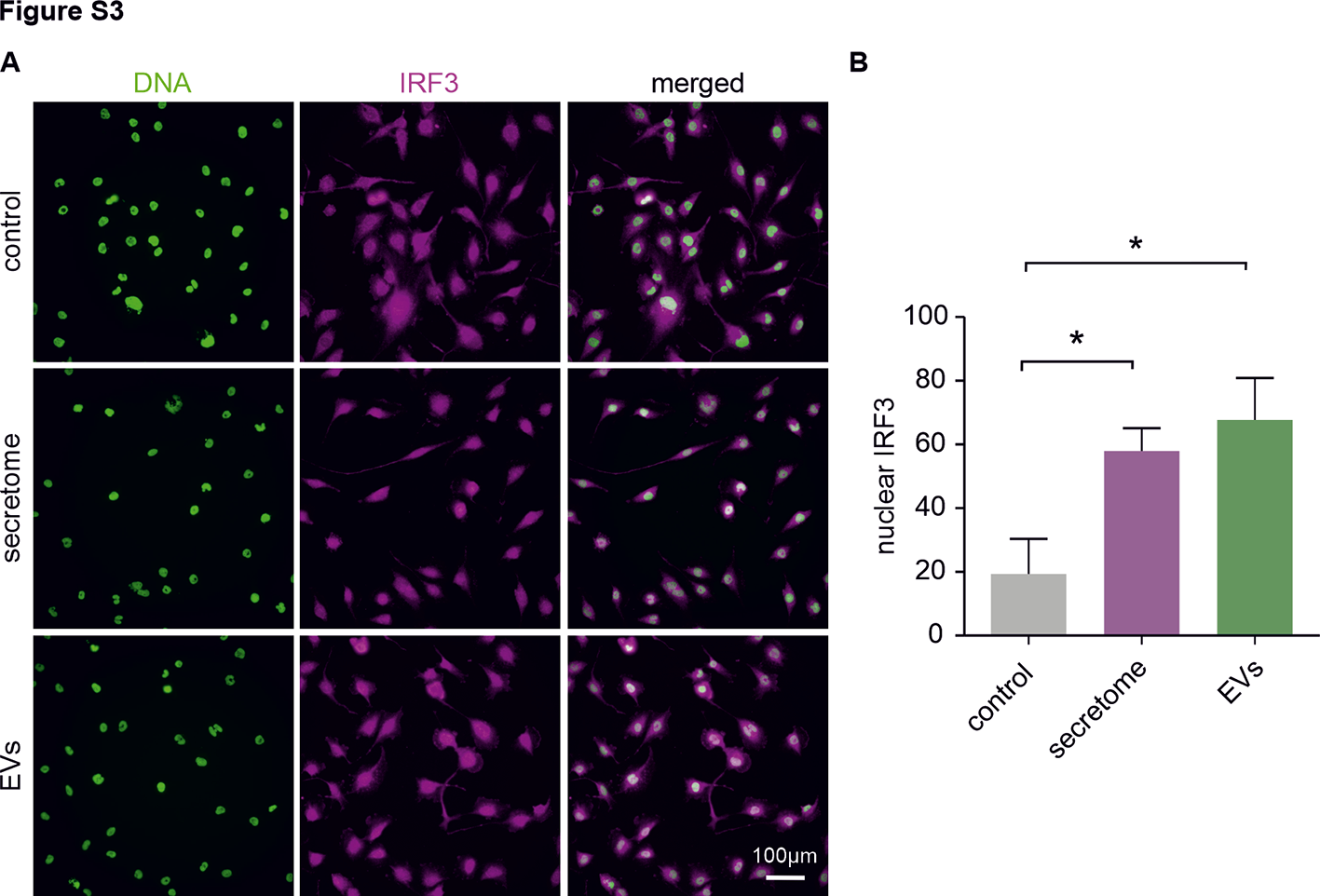

Supplement: Supplementary file 5 — (PNG 529 KB) [file 12015_2026_11133_Fig10_ESM.png]

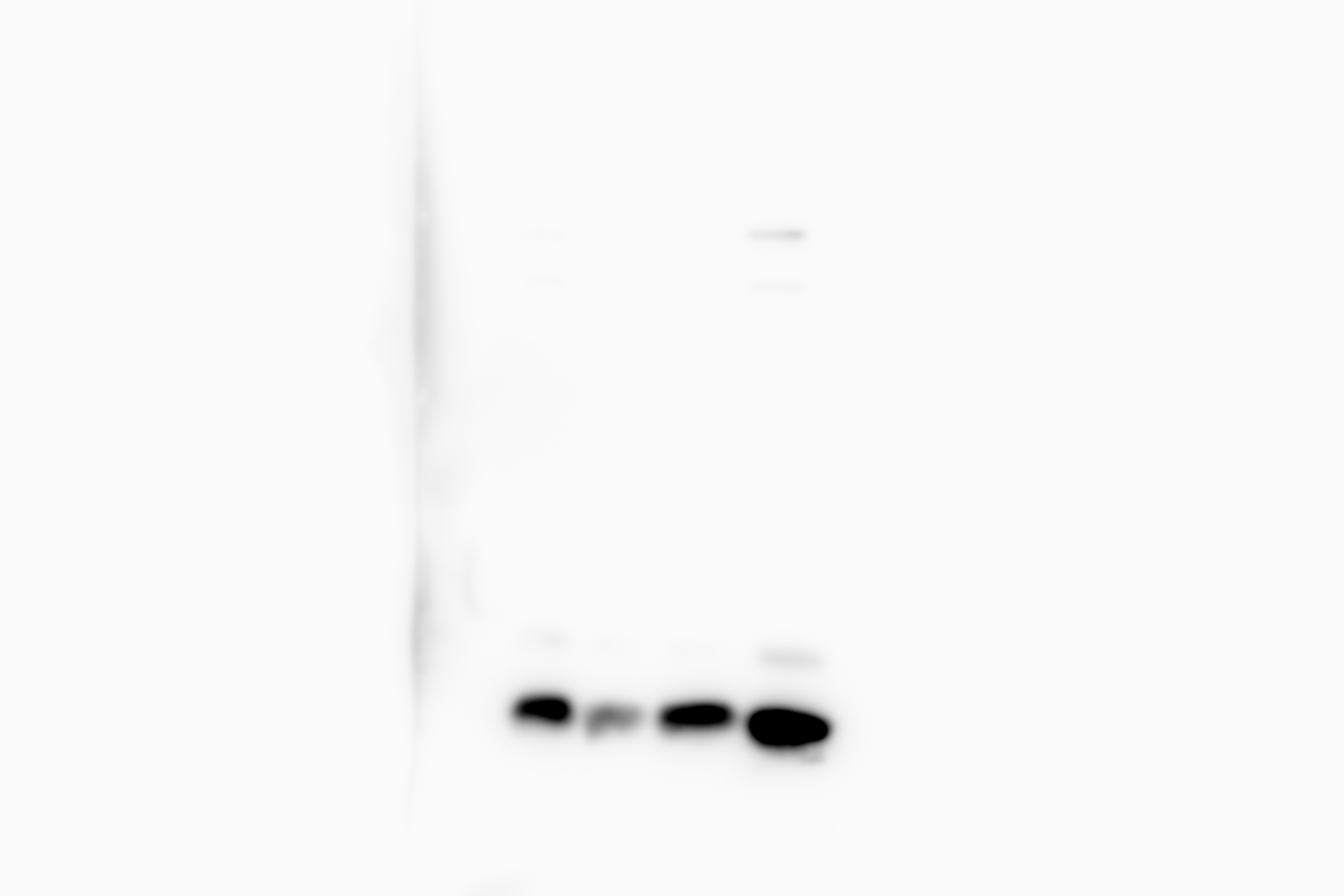

Supplement: Supplementary file 6 — NCSC secretomes and sEVs induce IRF3 nuclear translocation. (A) Representative spinning disc confocal microscopy images showing subcellular localisation of total IRF3 (magenta) in U251 cells treated with control, secretome, or sEVs. Nuclear translocation was evident in treated cells. Cell nuclei are counterstained with DAPI (green). Scale bar represents 100 μm. (B) Quantitative analysis showing significant enhancement of nuclear IRF3 translocation by both secretomes and sEVs compared to control conditions. Data represent mean ± SEM from three independent experiments. *p < 0.05, **p < 0.01. High Resolution Image (TIF 1.69 MB) [file 12015_2026_11133_MOESM3_ESM.tif]

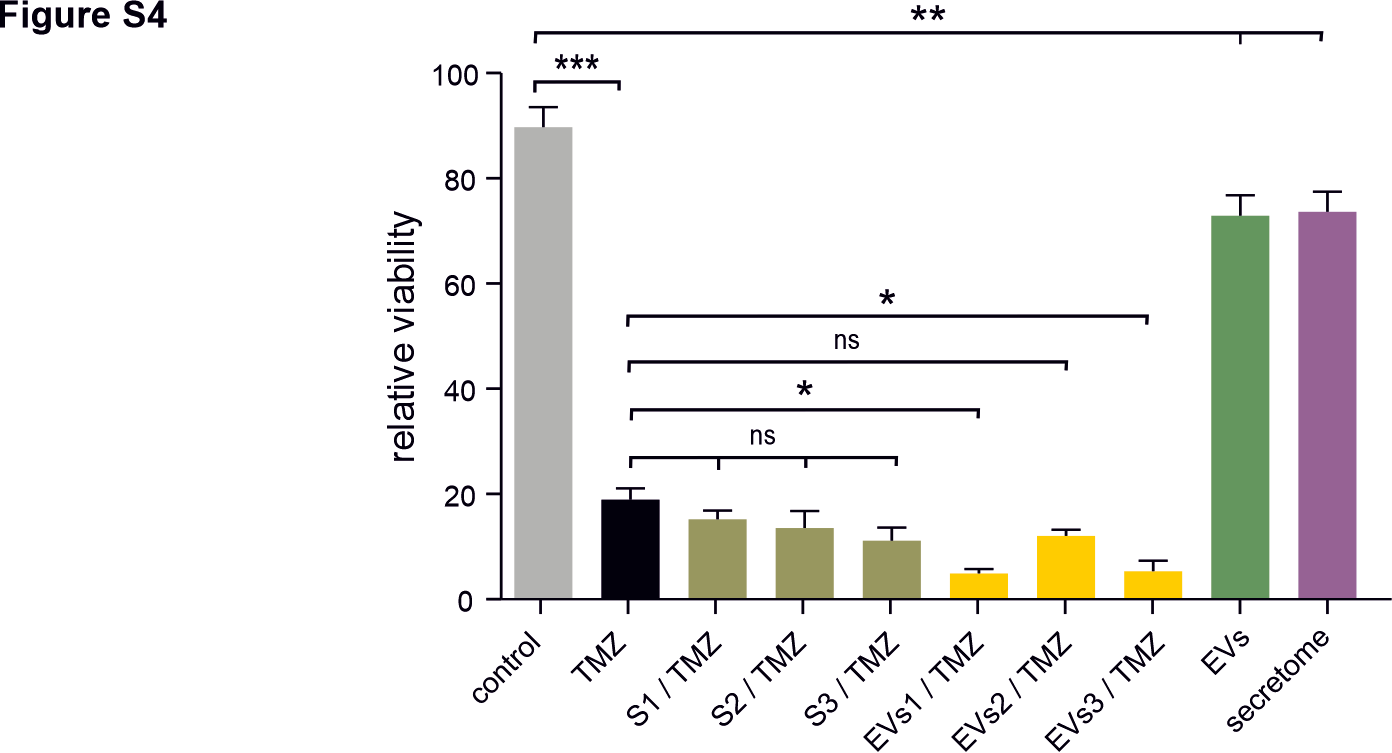

Supplement: Supplementary file 7 — (PNG 30.3 KB) [file 12015_2026_11133_Fig11_ESM.png]

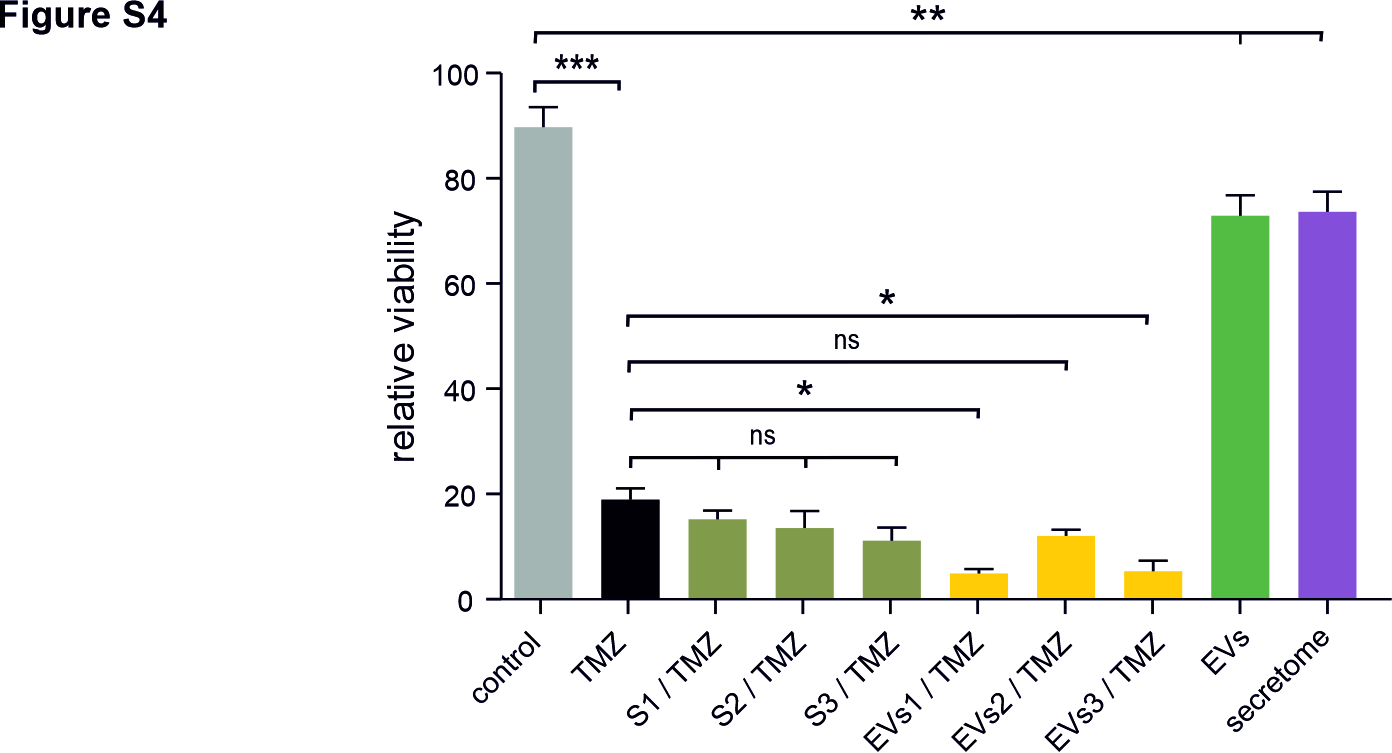

Supplement: Supplementary file 8 — Dual NF-κB gene reporter assays and quadruple NF-κB/IRF3 gene reporter assays confirm inflammatory rebalancing by NCSC secretomes and sEVs. A. Schematic representation of the dual NF-κB reporter system. B-D. The activity of the NF-κB was robustly activated by the pro-inflammatory factors TNF-α, IL-1β, and IL-6. This pro-inflammatory response was reduced after co-exposure to the respective pro-inflammatory factors and NCSC secretomes or EVs. E. Schematic of quadruple NF-κB/IRF3 reporter system allowing simultaneous pathway monitoring. F-G. Quadruple reporter analysis demonstrated TNF-α-mediated activation of NF-κB without IRF3 response, with a significant reduction after co-exposure to secretome (donor 3)/sEVs. Both secretomes and sEVs substantially increased IRF3 activity compared to control levels. Data represent mean ± SEM from three independent experiments. *p < 0.05, **p < 0.01, ***p < 0.001. High Resolution Image (TIF 161 KB) [file 12015_2026_11133_MOESM4_ESM.tif]

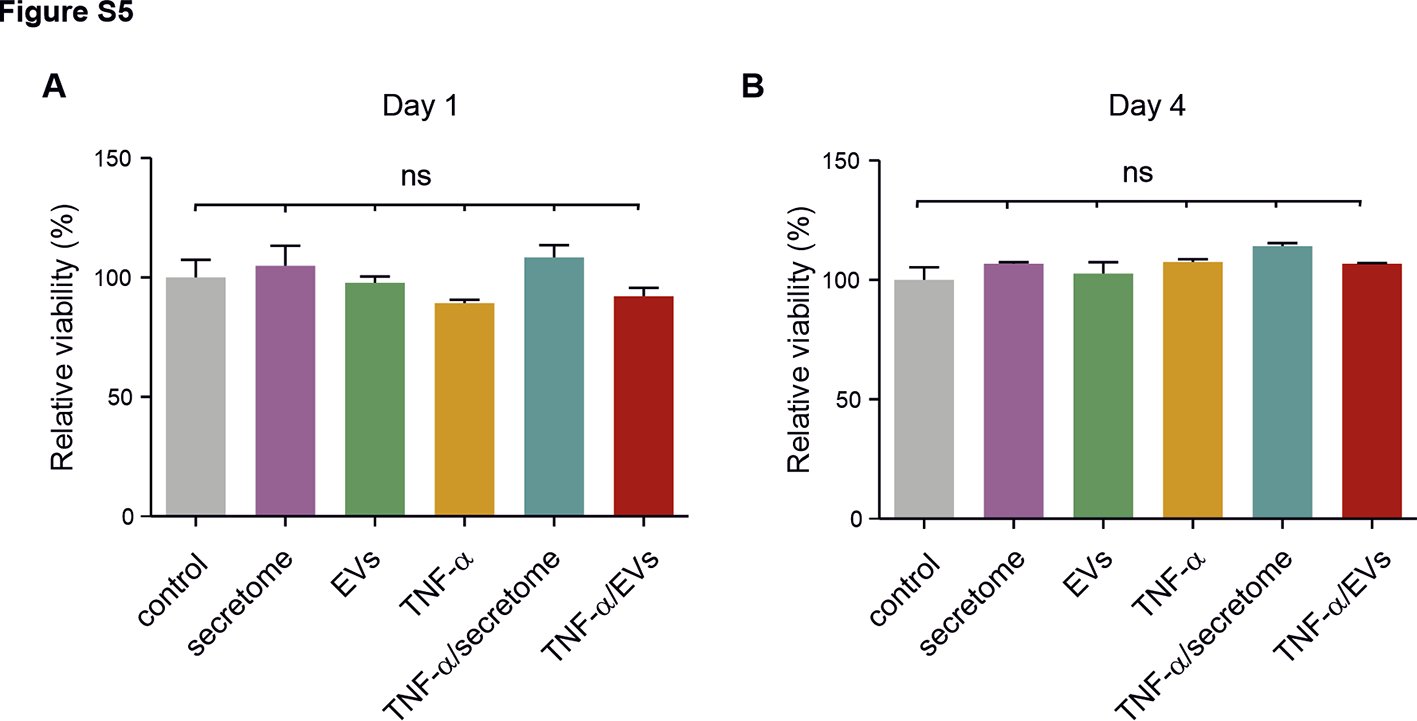

Supplement: Supplementary file 9 — (PNG 92.7 KB) [file 12015_2026_11133_Fig12_ESM.png]

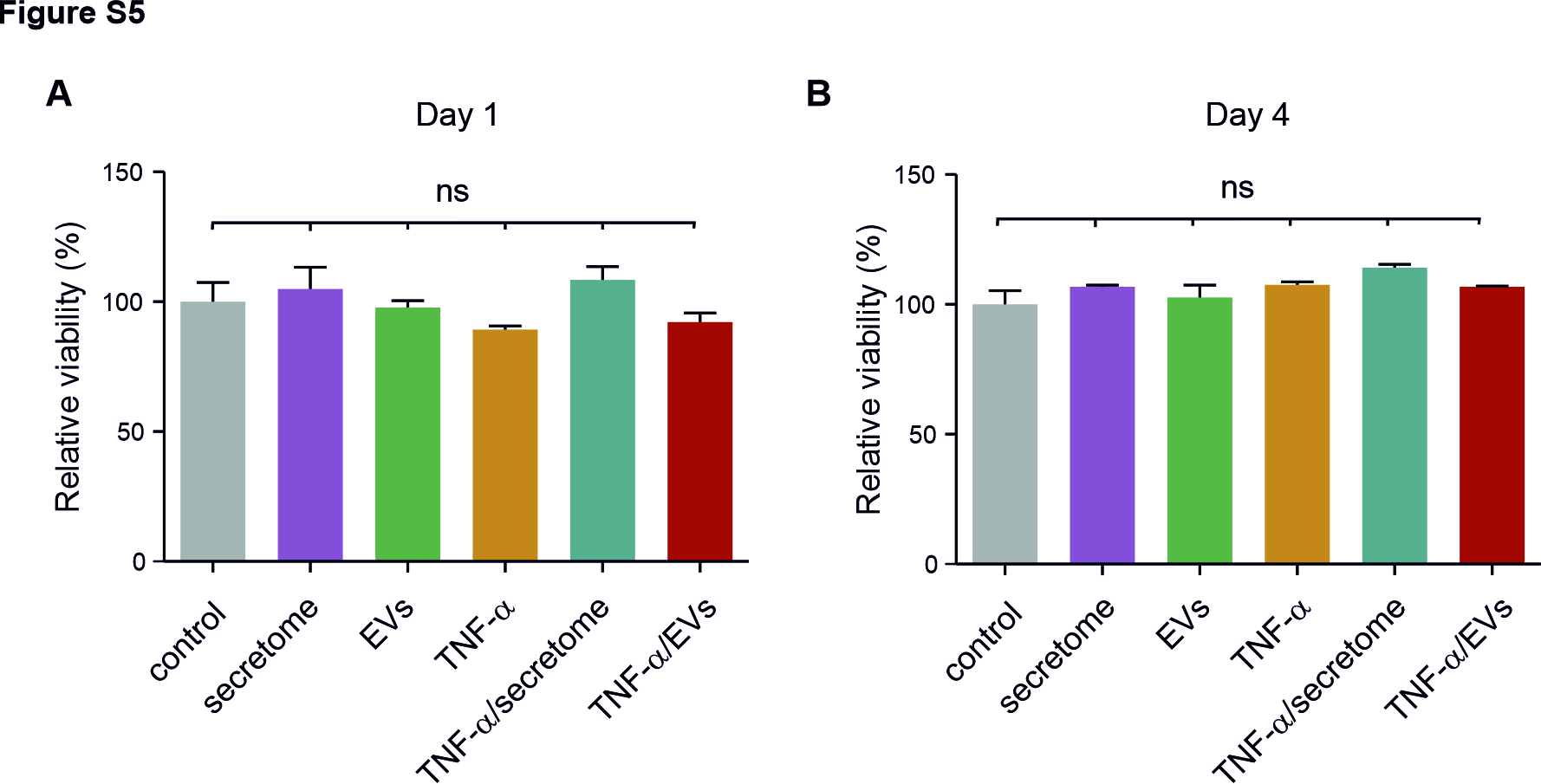

Supplement: Supplementary file 10 — NCSC products increase chemosensitivity to TMZ. XTT viability assay demonstrated TMZ-induced cytotoxicity and increased chemosensitivity with sEV from donors 1 and 3. Both secretomes and sEVs alone significantly reduce viability compared to controls. High Resolution Image (TIF 262 KB) [file 12015_2026_11133_MOESM5_ESM.tif]
